# Mapping the Thematic Structure and Evolution of AmpC β-Lactamase Research: A Bibliometric Analysis

**DOI:** 10.3390/antibiotics15080777

**Published:** 2026-08-13

**Authors:** Ahmet Doğukan Bayrak, Okan Derin

**Affiliations:** 1 Department of Infectious Diseases and Clinical Microbiology, Şişli Hamidiye Etfal Training and Research Hospital, 34396 Istanbul, Türkiye; okanderin@gmail.com; 2 Epidemiology Doctorate Program, Graduate School of Health Sciences, Istanbul Medipol University, 34815 Istanbul, Türkiye

**Keywords:** AmpC β-lactamases, bibliometrics, antimicrobial resistance, beta-lactamases, *Enterobacterales*, resistance ecology

## Abstract

Background: AmpC β-lactamases are complex resistance determinants due to inducible chromosomal expression, stable derepression, plasmid-mediated dissemination, potential for co-resistance, and diagnostic interpretation challenges. Nevertheless, the scientific production, citation structure, and thematic evolution of the AmpC β-lactamase literature have not been comprehensively mapped. This study aimed to evaluate this literature using bibliometric methods. Methods: Original articles and reviews indexed in SCI-E and ESCI within the Web of Science Core Collection were evaluated when “AmpC” appeared in the title field. After an additional microbiological filter, 1183 documents published between 1980 and 2026 were included. Analyses were performed using R, bibliometrix, and Biblioshiny. Results: Scientific production increased from the 2000s onward. Antimicrobial Agents and Chemotherapy and Journal of Antimicrobial Chemotherapy were among the most productive and most locally cited sources, while the United States ranked first in publication output and total citation impact. The most frequent keywords were *Escherichia coli*, ESBL, *Klebsiella pneumoniae*, and *Enterobacterales*. Conceptual analyses showed that the literature was structured around chromosomal AmpC biology, plasmid-mediated dissemination, resistance ecology intersecting with ESBLs, a cluster interpretable as a One Health axis, and clinical management themes. Trend topic analysis showed that multidrug resistance, carbapenemase, and virulence have become more visible in recent years. Conclusions: AmpC β-lactamase literature has broadened from an earlier emphasis on chromosomal resistance biology toward a multidimensional research field encompassing plasmid-mediated dissemination, resistance ecology, One Health-related themes, and clinical management. The recent prominence of concepts related to multidrug resistance phenotypes, the concurrent dissemination of resistance determinants, and pathogen virulence may suggest that the field is increasingly being addressed within a broader antimicrobial resistance context.

## 1. Introduction

Antimicrobial resistance remains one of the major challenges in modern infectious diseases practice, particularly because of mechanisms that limit the activity of β-lactam antibiotics against Gram-negative bacteria [1]. Among these resistance mechanisms, AmpC β-lactamases occupy an important position and are classified as molecular class C and functional group 1 cephalosporinases in contemporary β-lactamase classification schemes [2]. These enzymes are clinically distinctive because of their hydrolytic activity against extended-spectrum cephalosporins and cephamycins, their limited susceptibility to older/classical β-lactamase inhibitors, and their capacity for inducible expression in certain bacterial species [3]. AmpC production may be chromosomally encoded and inducible, may result in constitutively high-level expression through stable derepression, or may disseminate across different species via plasmids [4,5,6]. This biological diversity translates into diagnostic, epidemiological, and therapeutic complexity, making AmpC β-lactamases a clinically important resistance mechanism [3,7].

Nevertheless, the developmental trajectory, scientific structure, and thematic evolution of the AmpC β-lactamase literature have not been comprehensively mapped. Consistent with this gap, searches of the Web of Science Core Collection, Scopus, and PubMed using “AmpC” and terms related to bibliometric analysis did not identify any eligible study specifically mapping the AmpC β-lactamase literature. Although bibliometric analyses have examined adjacent β-lactam resistance fields, including ESBL-producing Escherichia coli and carbapenem-resistant Enterobacteriaceae [8,9], none has specifically focused on AmpC β-lactamases. Existing narrative reviews have synthesized the molecular classification, diagnostic challenges, epidemiology, and therapeutic implications of AmpC β-lactamases [3,4]. However, these reviews do not quantitatively demonstrate how the field has grown over time, which authors, countries, and journals have shaped the literature, which publications have had the greatest influence, or which research themes have emerged during different periods. In contrast, the AmpC literature has evolved into a multilayered scientific field extending from chromosomal resistance biology to plasmid-mediated dissemination, from overlapping ESBL and AmpC resistance contexts to clinical treatment decisions [3,5]. Therefore, this field warrants complementary evaluation through bibliometric and science mapping methods.

Bibliometric analyses allow the quantitative evaluation of scientific production, country-level contributions, journal distribution, highly cited publications, and conceptual themes within a defined research field. This approach is particularly useful for macro-level assessment of studies that have diversified into different subthemes over long periods and acquired an interdisciplinary character [10]. One of the tools used to conduct such analyses is bibliometrix, a comprehensive R-based science mapping package that enables descriptive, conceptual, and structural analysis of the scientific literature [11]. In this context, the AmpC β-lactamase literature represents a suitable and meaningful research area for bibliometric analysis because it encompasses both microbiological diversity and clinically relevant resistance phenotypes.

In this study, we performed a bibliometric analysis of the AmpC β-lactamase literature indexed in the Web of Science Core Collection. By evaluating annual scientific production trends, leading journals, country contributions, influential publications, citation patterns, and keyword-based conceptual structures, we aimed to characterize the historical development and thematic evolution of the field over time.

## 2. Results

### 2.1. Dataset Characteristics and Data Coverage

The final search strategy retrieved 1183 documents published between 1980 and 2026. Of the 15 publications included in the predefined benchmark seed set, 12 were retrieved, corresponding to an overall recall of 80.0%. Axis-stratified recall was 80.0% for the foundational and diagnostic axis (4/5), 83.3% for the clinical and therapeutic axis (5/6), and 75.0% for the One Health and food-chain axis (3/4). The three non-retrieved publications comprised a broad review of β-lactamase classification, a general IDSA guidance document on antimicrobial-resistant Gram-negative infections, and one study assigned to the One Health and food-chain axis. These data are presented in Table S1.

Of the 91 unique candidate publications identified through the selected Web of Science research areas and categories and the author-based cross-check, 46 were classified within the structural and enzymological research axis after manual title and abstract screening. These publications primarily addressed AmpC enzymology, protein structure, inhibitor design, molecular docking, or virtual screening and represented 3.9% of the final corpus. The publications included in this axis are presented in Table S2.

The retrieved documents were published in 328 different sources and included 5332 authors. The annual growth rate was 7.67%. In an additional analysis restricted to complete publication years, the compound annual growth rate was 9.48% for 1980–2025. Sensitivity analyses using alternative decennial base years yielded growth rates of 10.15% for 1990–2025 and 5.10% for 2000–2025, indicating that the estimate was sensitive to the low publication counts in the early years of the series. The average number of citations per document was 35.21, and the average number of co-authors per document was 6.31. The rate of international co-authorship was 22.57%. The general characteristics of the dataset are presented in Table S3.

The missing data analysis revealed no missing values for authors, document type, source, language, publication year, title, or total citations. The proportion of missing data ranged from 0.08% to 6.51% across the cited references, abstracts, corresponding author, DOIs, and Keywords Plus fields. The highest proportion of missing data was observed in the author keywords, with 358 missing records (30.26%). These data are presented in Table S4.

Keyword coverage, defined as the presence of at least one term in either the author keywords or Keywords Plus field, increased from 70.7% in 1980–1999 to 98.6–99.6% across the post-2000 periods. Overall, 34 documents (2.87%) contained neither author keywords nor Keywords Plus. These data are presented in Table S5.

### 2.2. Scientific Production and Source Landscape

**Figure 1:**
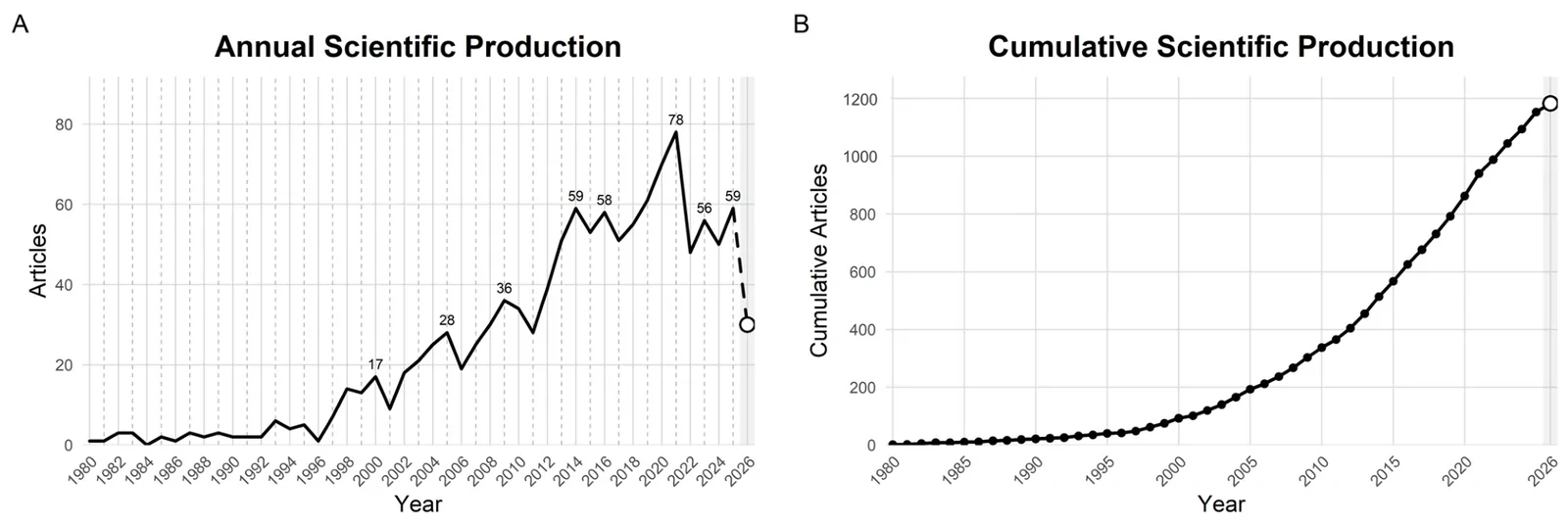
Temporal evolution of AmpC β-lactamase research: Annual and cumulative scientific production. (**A**) Annual number of publications on AmpC β-lactamase research during the study period. (**B**) Cumulative scientific output of AmpC β-lactamase research based on records retrieved from the Web of Science Core Collection. The gray shaded area denotes the partial 2026 period, while the dashed segment and open circles indicate partial 2026 data up to 15 June 2026.

The annual scientific production curve showed that the number of publications remained low between 1980 and 1996. From 1997 onward, scientific output increased markedly, despite year-to-year fluctuations, and from 2003 onward the annual count mostly exceeded 20 publications. After 2013, the publication volume reached a higher level and exceeded 50 publications in several years. The highest annual count was observed in 2021, with 78 publications. Between 2022 and 2025, annual output ranged from 48 to 59 publications, while 30 publications were indexed in 2026 by 15 June 2026 (Figure 1A). The cumulative growth curve showed that publication accumulation accelerated particularly from the early 2000s onward, reaching a total of 1183 publications by the search date (Figure 1B).

**Figure 2:**
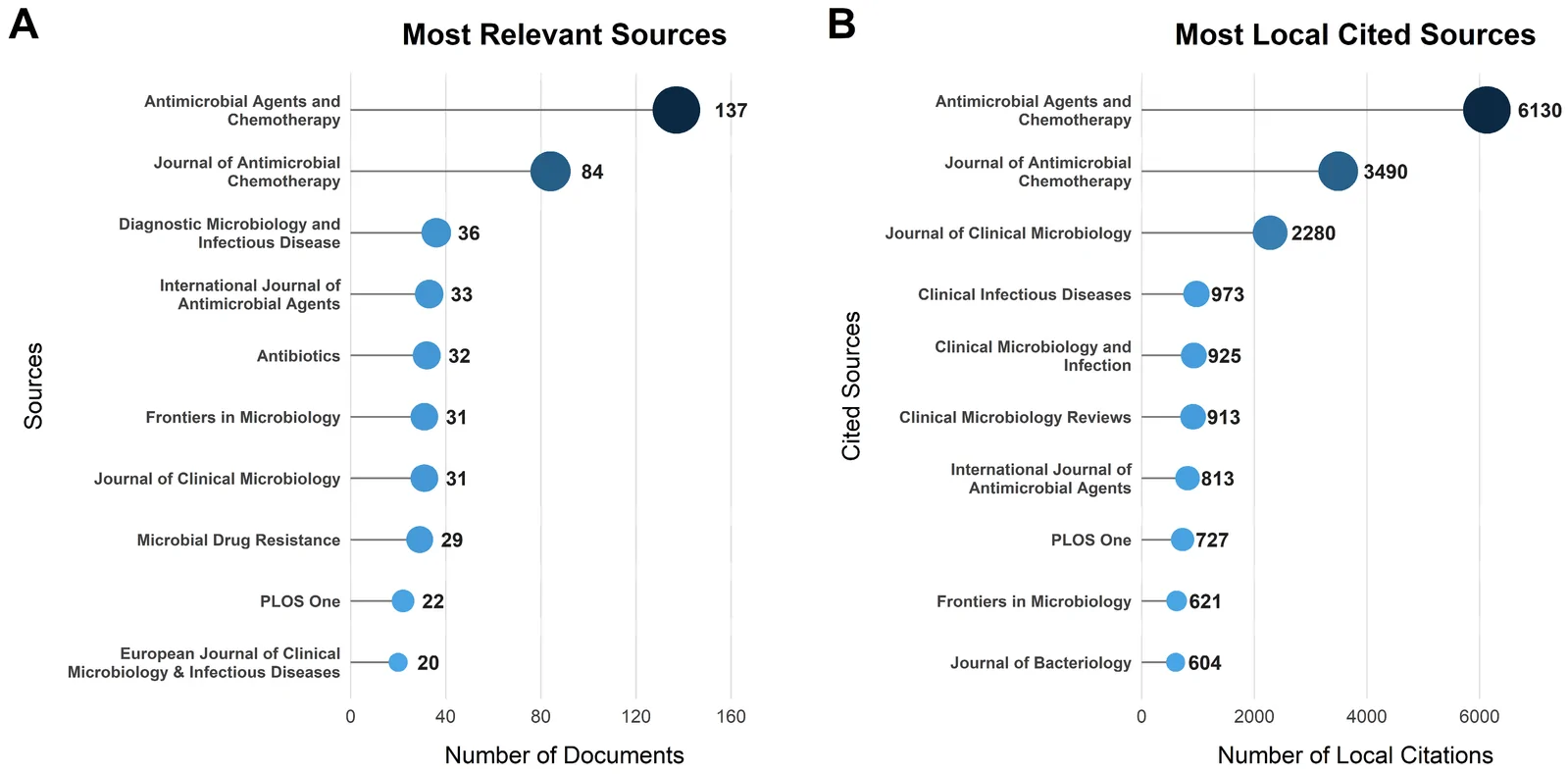
Most relevant and most locally cited sources in the AmpC β-lactamase literature. (**A**) Leading journals according to publication output within the analyzed AmpC β-lactamase dataset. (**B**) Leading journals according to local citation count. Local citations refer to citations received from documents included in the present bibliometric dataset.

In the source analysis, *Antimicrobial Agents and Chemotherapy* and *Journal of Antimicrobial Chemotherapy* ranked first and second, respectively, both by publication count and by local citations within the dataset. Among the most productive publication sources, these two journals were followed by *Diagnostic Microbiology and Infectious Disease*, *International Journal of Antimicrobial Agents*, and *Antibiotics (Basel),* forming the top five sources (Figure 2A). All ten of the most productive sources were indexed in SCI-E, and eight were ranked in Q1 or Q2. Their 2025 JIF values and category-specific quartiles are provided in Table S6.

Comparison of the top 10 sources showed that six journals overlapped between the lists of the most productive sources by publication count and the most influential sources by local citation count. In contrast, *Clinical Infectious Diseases, Clinical Microbiology and Infection, Clinical Microbiology Reviews,* and *Journal of Bacteriology* were not among the top 10 by publication count but appeared in the latter list based on local citations (Figure 2B).

**Figure 3:**
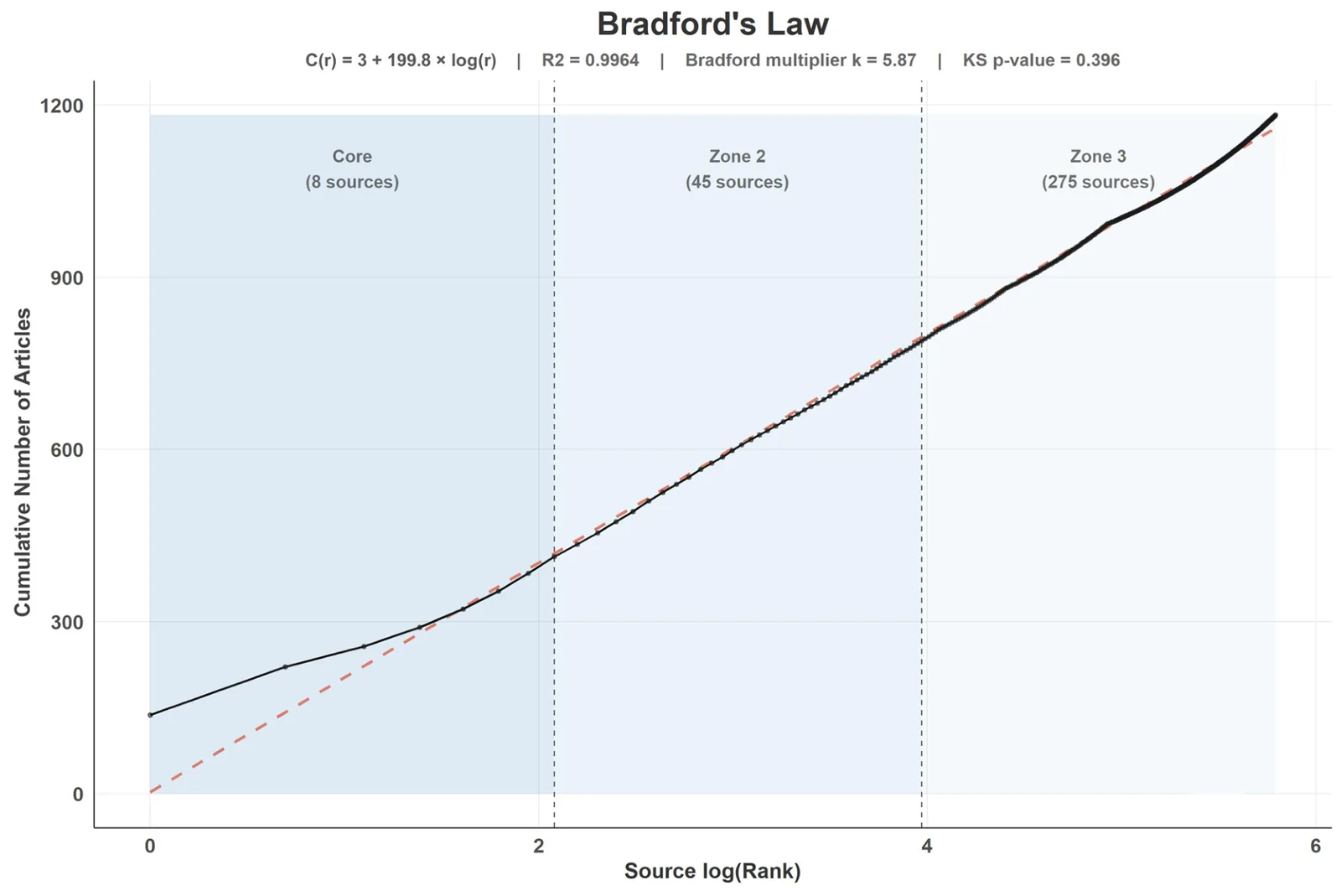
Bradford’s Law distribution of publication sources. The 328 sources were divided into a core zone, Zone 2, and Zone 3, each contributing approximately one-third of the publications. The solid black line represents the observed cumulative distribution of articles across ranked sources, the red dashed line represents the fitted Bradford model, and the vertical dashed lines indicate the boundaries between the Bradford zones.

Bradford’s Law analysis showed that the literature was concentrated in a small core set of sources and then dispersed across progressively larger zones. The core zone comprised 8 sources, followed by 45 sources in Zone 2 and 275 sources in Zone 3, with each zone contributing approximately one-third of the total publications (Figure 3). The observed source distribution showed a close fit to Bradford’s model (Bradford multiplier k = 5.87; R^2^ = 0.9964; KS *p* = 0.396).

### 2.3. Author and Institutional Contributions

**Figure 4:**
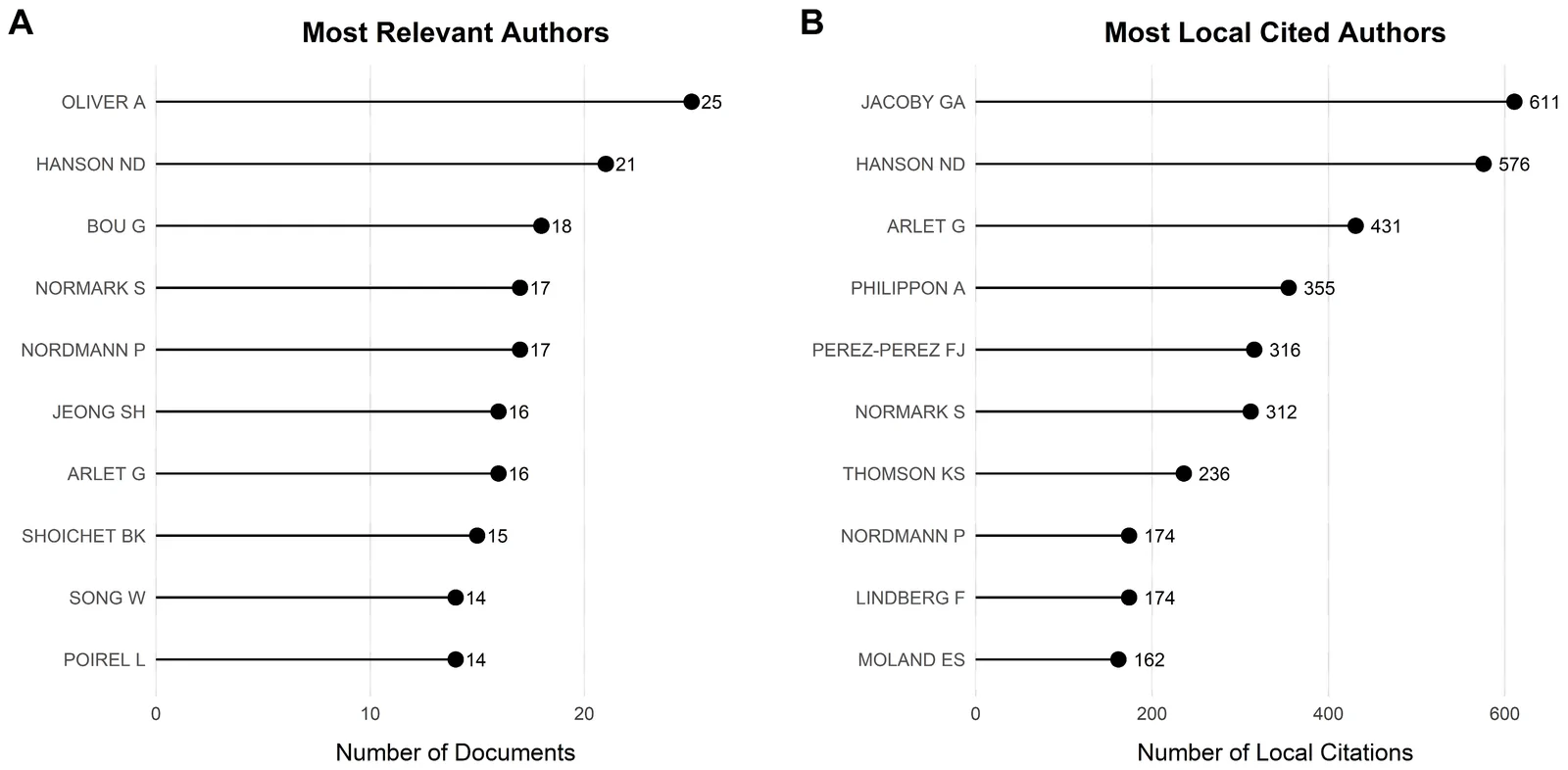
Most relevant and most locally cited authors in the AmpC β-lactamase literature. (**A**) Leading authors according to publication output within the analyzed AmpC β-lactamase dataset. (**B**) Leading authors according to local citation count. Local citations refer to citations received from documents included in the present bibliometric dataset.

At the author level, publication productivity and local citation impact within the dataset did not fully overlap. Oliver A was the most productive author by publication count, whereas Jacoby GA ranked first by local citations. A comparison of the top 10 author lists showed that Hanson ND, Arlet G, Normark S, and Nordmann P appeared on both lists (Figure 4).

**Figure 5:**
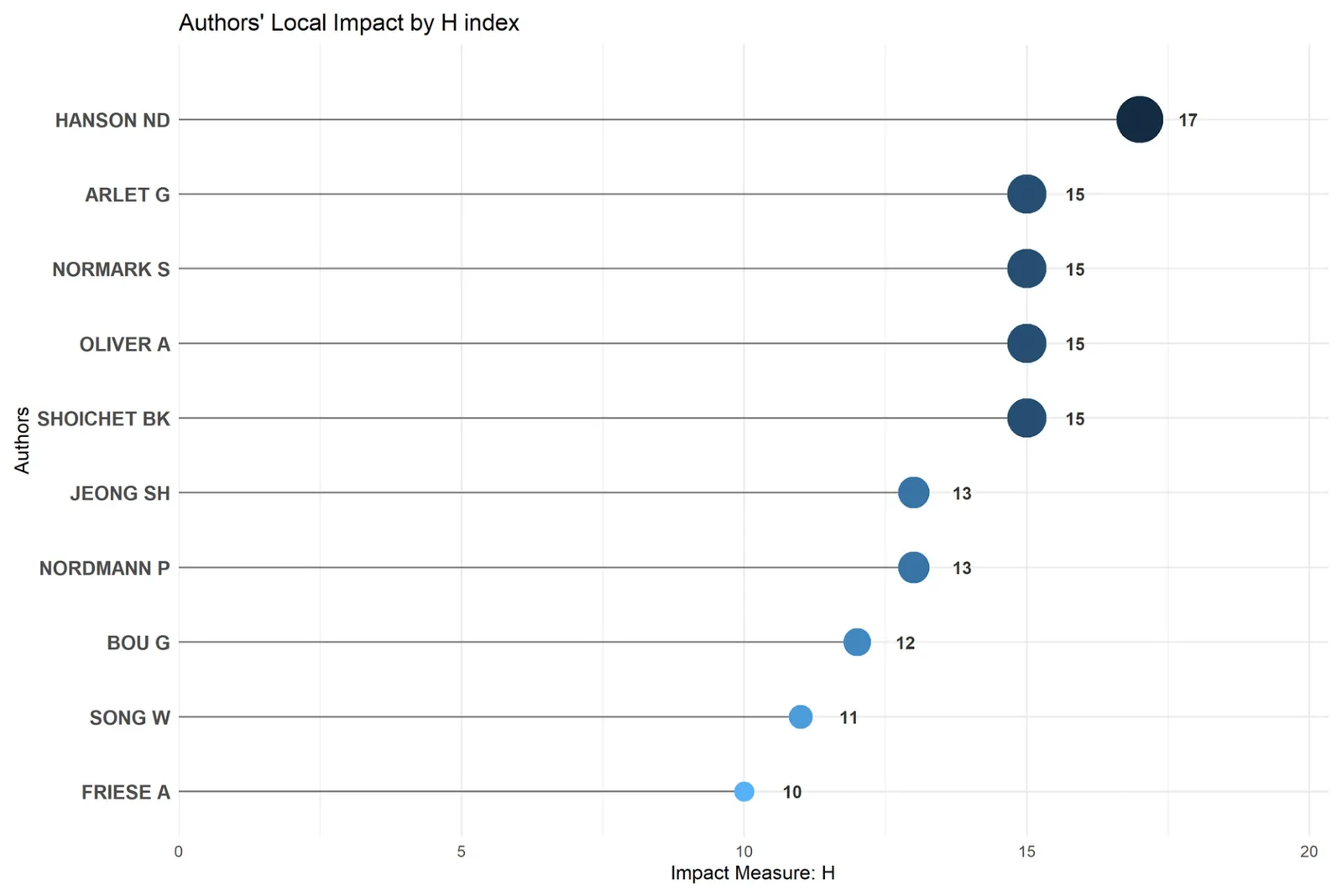
Authors’ local impact by h-index. Local h-index values were calculated using citations within the analyzed dataset.

Authors’ local impact was further evaluated using the h-index calculated within the dataset. Hanson ND had the highest local h-index (h = 17), followed by Arlet G, Normark S, Oliver A, and Shoichet BK, each with an h-index of 15. Jeong SH and Nordmann P both had an h-index of 13 (Figure 5).

**Figure 6:**
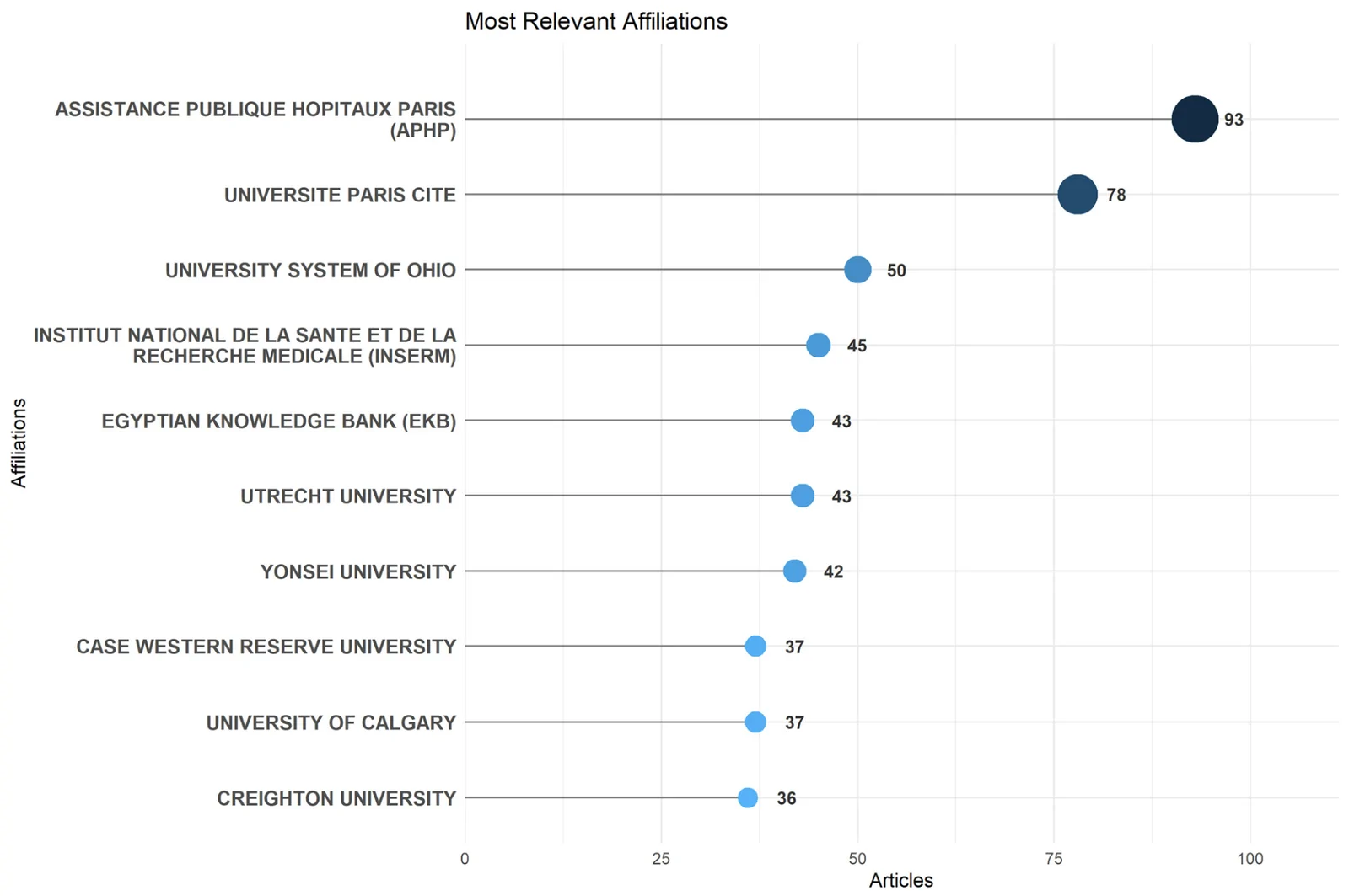
Most relevant institutional affiliations. The top 10 affiliations were ranked by the number of articles in the dataset.

At the institutional level, Assistance Publique–Hôpitaux de Paris was the most productive affiliation with 93 articles, followed by Université Paris Cité with 78 and the University System of Ohio with 50 articles (Figure 6).

### 2.4. Country Contributions and International Collaboration

**Figure 7:**
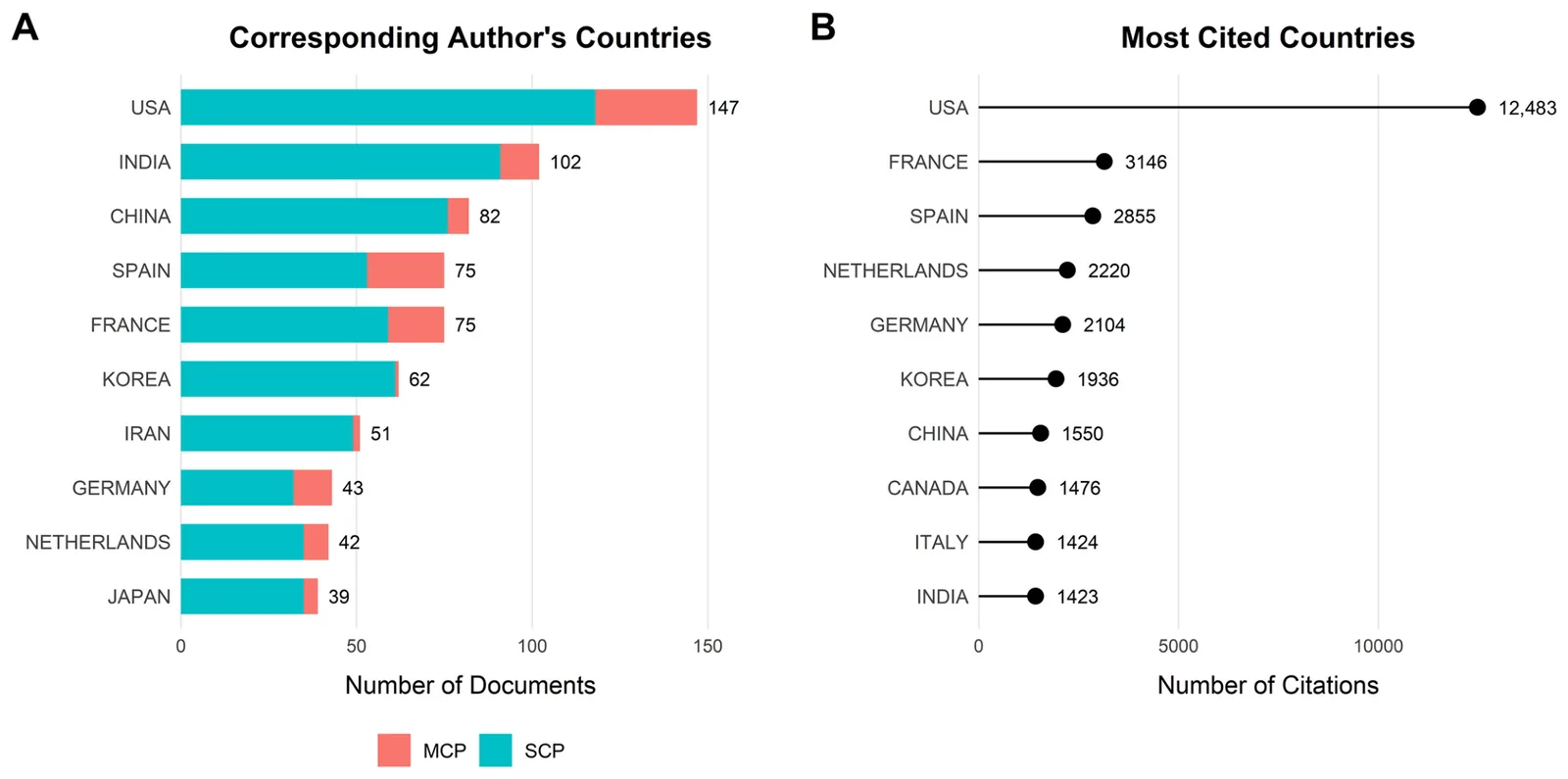
Country-level productivity and citation impact in the AmpC β-lactamase literature. (**A**) The most productive countries based on the corresponding author’s affiliation, with publications classified as single-country publications (SCP) or multiple-country publications (MCP). (**B**) The most cited countries based on total citations received by documents in the analyzed AmpC β-lactamase dataset.

In the distribution of publications by corresponding author country, the highest number of documents originated from the United States, followed by India, China, Spain, and France. Single-country publications were predominant across all countries, although the contribution of multiple-country publications was more evident for the United States, Spain, and France (Figure 7A). The ranking changed when countries were assessed by total citations: the United States ranked first by a wide margin (12,483 citations), followed by France (3146 citations) and Spain (2855 citations), which ranked second and third, respectively (Figure 7B).

**Figure 8:**
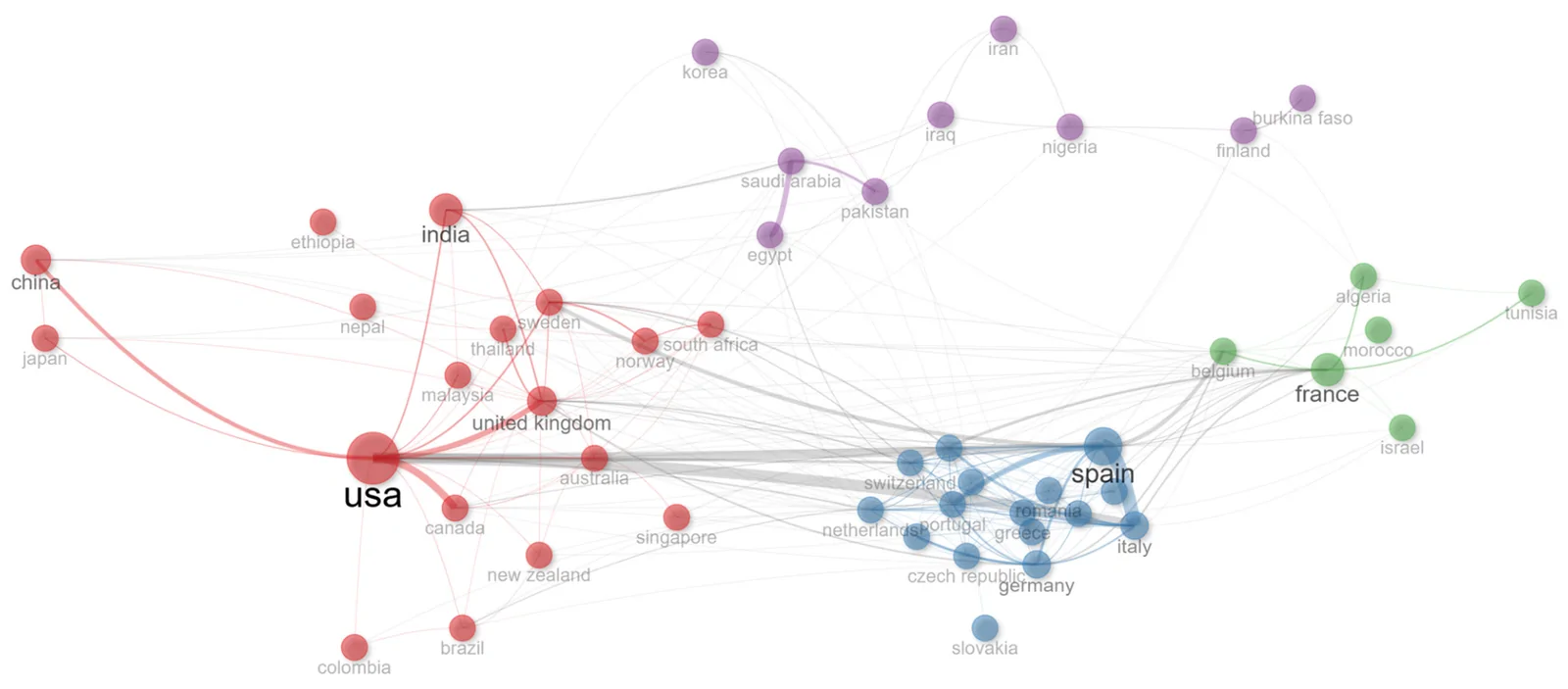
Country Collaboration Network in AmpC β-Lactamase Research. Node size reflects publication output, edge thickness indicates collaboration strength, and colors represent collaboration clusters.

The country-level collaboration network showed a clustered structure centered on a limited number of highly connected countries. The United States formed the largest node and had extensive links with countries across Europe, Asia, and other regions. Spain and France also represented prominent hubs within predominantly European collaboration clusters, whereas most other countries appeared as smaller nodes with fewer and less dense international connections (Figure 8).

### 2.5. Citation Impact of Documents and References

**Figure 9:**
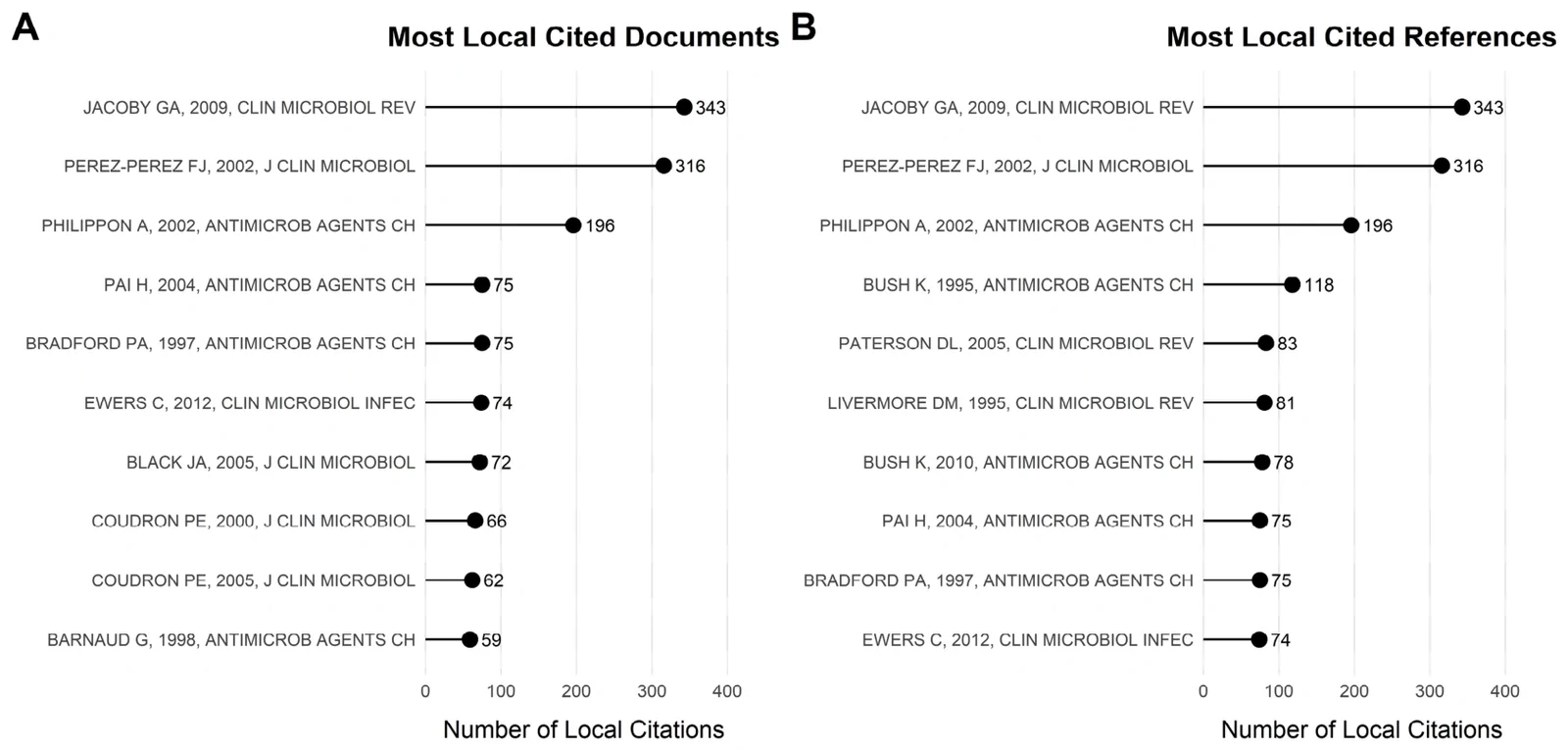
Most locally cited documents and references in the AmpC β-lactamase literature. (**A**) Most locally cited documents based on citations received from records included in the analyzed AmpC β-lactamase dataset [3,4,12,13,14,15,16,17,18,19]. (**B**) Most frequently cited references in the reference lists of the included documents [2,3,4,12,13,14,15,20,21,22]. Unlike Panel A, these references may include influential works that were not themselves included in the analyzed dataset.

A comparison between the most locally cited documents and the references most frequently cited in the bibliographies of the included articles revealed that the top three studies were identical (Figure 9). The leading work was the 2009 article by Jacoby GA, published in *Clinical Microbiology Reviews*, which received 343 local citations. This was followed by the 2002 article by Pérez-Pérez FJ in *Journal of Clinical Microbiology*, with 316 local citations, and the 2002 publication by Philippon A in *Antimicrobial Agents and Chemotherapy*, with 196 local citations.

### 2.6. Conceptual Structure and Thematic Evolution

**Figure 10:**
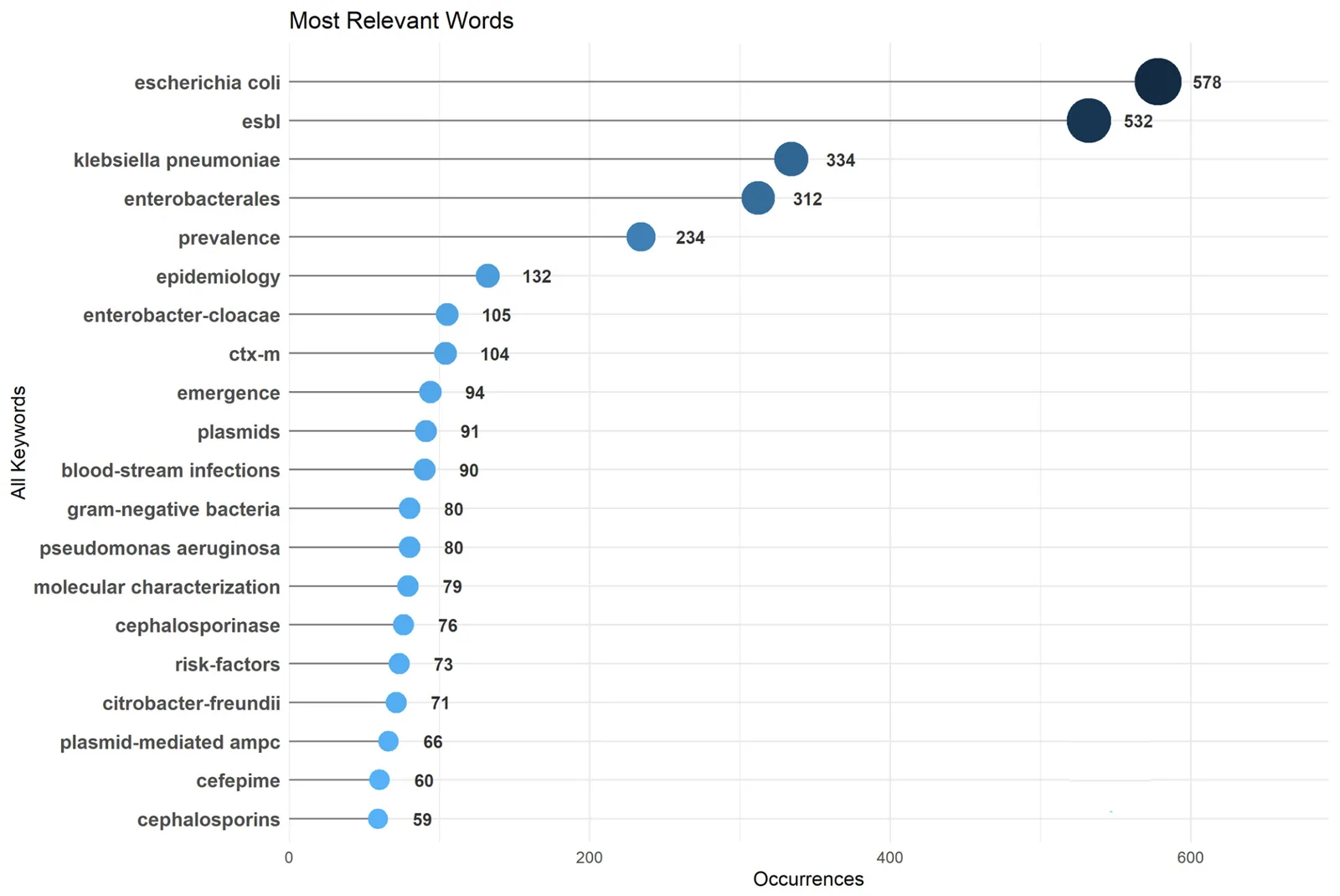
Most relevant words in the AmpC β-lactamase literature. The most frequent terms were identified from the All Keywords field after synonym standardization and stop-word removal.

Following synonym normalization and stop-word removal, the most frequent keywords were *Escherichia coli* (n = 578), ESBL (n = 532), *Klebsiella pneumoniae* (n = 334), Enterobacterales (n = 312), prevalence (n = 234), and epidemiology (n = 132). The 20 most frequently occurring keywords are presented in Figure 10.

In the expanded search sensitivity corpus, 19 of the 20 highest-ranking terms in the primary analysis were retained (95.0%), and the five highest-ranking terms remained in identical positions. Of the 19 shared terms, 18 (94.7%) changed by no more than two positions, whereas “cephalosporinase” moved from 15th to 12th place. “Cephalosporins” was replaced by “expression” (Figure S1).

**Figure 11:**
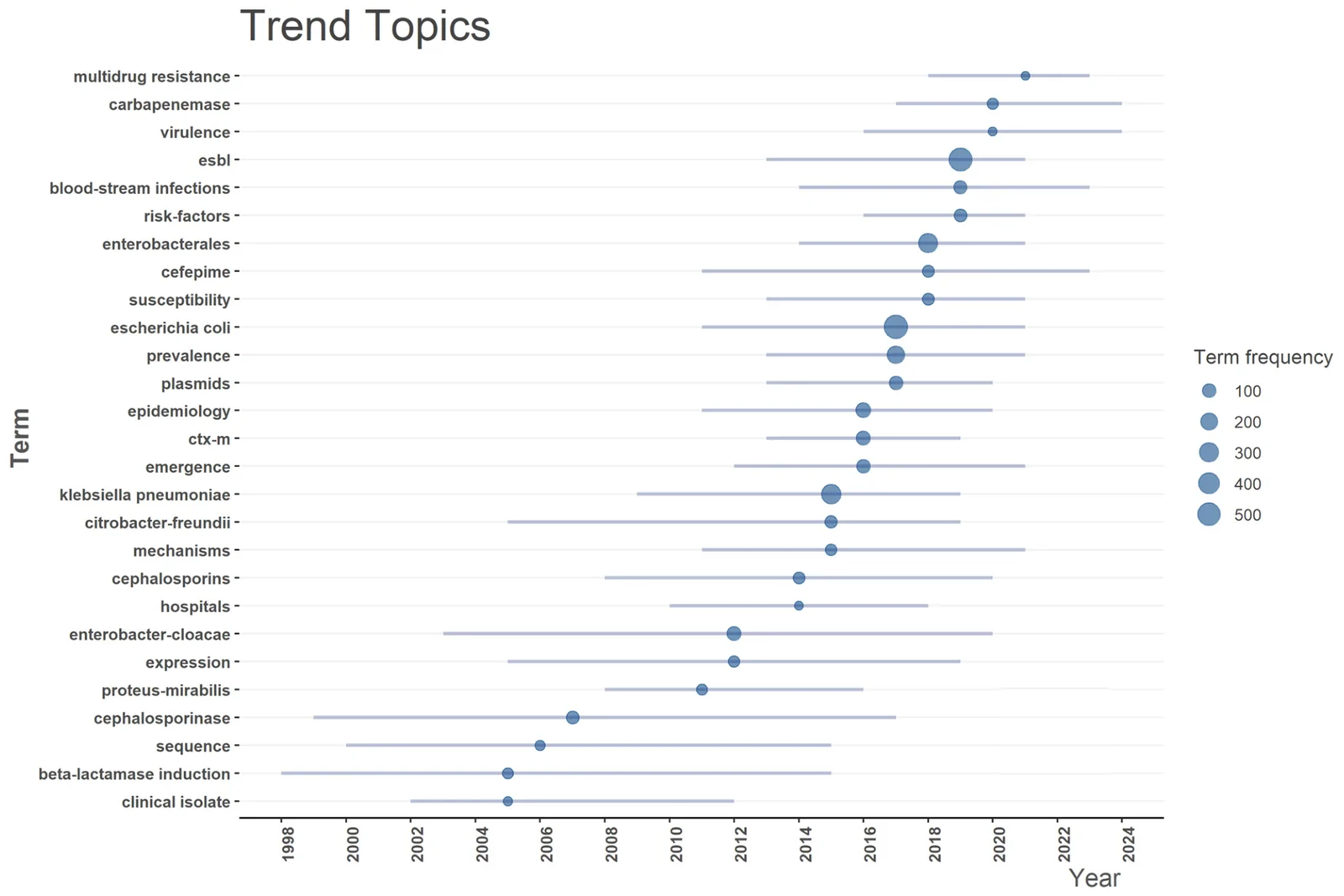
Trend topics in the AmpC β-lactamase literature. The figure shows the temporal distribution of the most frequent terms identified from the All Keywords field after synonym standardization and stop-word removal. Term size reflects frequency within the analyzed AmpC β-lactamase dataset.

The trend topic analysis evaluated the distribution of keywords over time. In the early 2000s, when publication activity began to increase, terms such as “clinical isolate,” “beta-lactamase induction,” “sequence,” and “cephalosporinase” were prominent. From 2010 onward, terms including *Proteus mirabilis*, “expression,” *Enterobacter cloacae*, “hospitals,” “cephalosporins,” “mechanisms,” and *Citrobacter freundii* became more visible. From 2015 onward, *Klebsiella pneumoniae*, “emergence,” CTX-M, “epidemiology,” “plasmids,” “prevalence,” and *E. coli* were increasingly represented. From 2018 onward, “susceptibility,” “cefepime,” *Enterobacterales*, “risk factors,” “bloodstream infections,” and ESBL became more prominent. From 2020 onward, “virulence,” “carbapenemase,” and “multidrug resistance” emerged as the most recent trend topics. The trend topic analysis is presented in Figure 11.

In the expanded search sensitivity corpus, 23 of the 27 trend topics displayed in the primary analysis were retained (85.2%), with a broadly similar temporal distribution. The three most recent trend topics—“virulence,” “carbapenemase,” and “multidrug resistance”—were retained, while “bloodstream infections” and “risk factors” remained centered around 2019. “Cefepime,” CTX-M, “mechanisms,” and “hospitals” were no longer displayed, whereas “animals,” *Pseudomonas aeruginosa*, “Gram-negative bacteria,” CMY-2, “outbreak,” *Serratia*, and “cloning” newly appeared among the displayed trend topics (Figure S2).

In the stop-word sensitivity analysis, 25 of the 27 trend topics displayed in the primary analysis were retained (92.6%). All six topics centered between 2019 and 2021—“multidrug resistance,” “carbapenemase,” “virulence,” ESBL, “bloodstream infections,” and “risk factors”—were retained. The reintroduced general terms “antibiotic resistance,” “infections,” “resistance,” and “genes” newly appeared, whereas “susceptibility” and “mechanisms” were no longer displayed (Figure S3).

In the author-keyword-only sensitivity analysis, all 10 displayed trend topics had median publication years of 2016 or later. Eight of the 10 displayed trend topics were also present in the primary All Keywords analysis. “Multidrug resistance,” “bloodstream infections,” and *Enterobacterales* were retained among the most recent topics, whereas “virulence” and “carbapenemase” were not displayed; CMY-2 and *Pseudomonas aeruginosa* appeared only in the author-keyword-only analysis (Figure S4).

**Figure 12:**
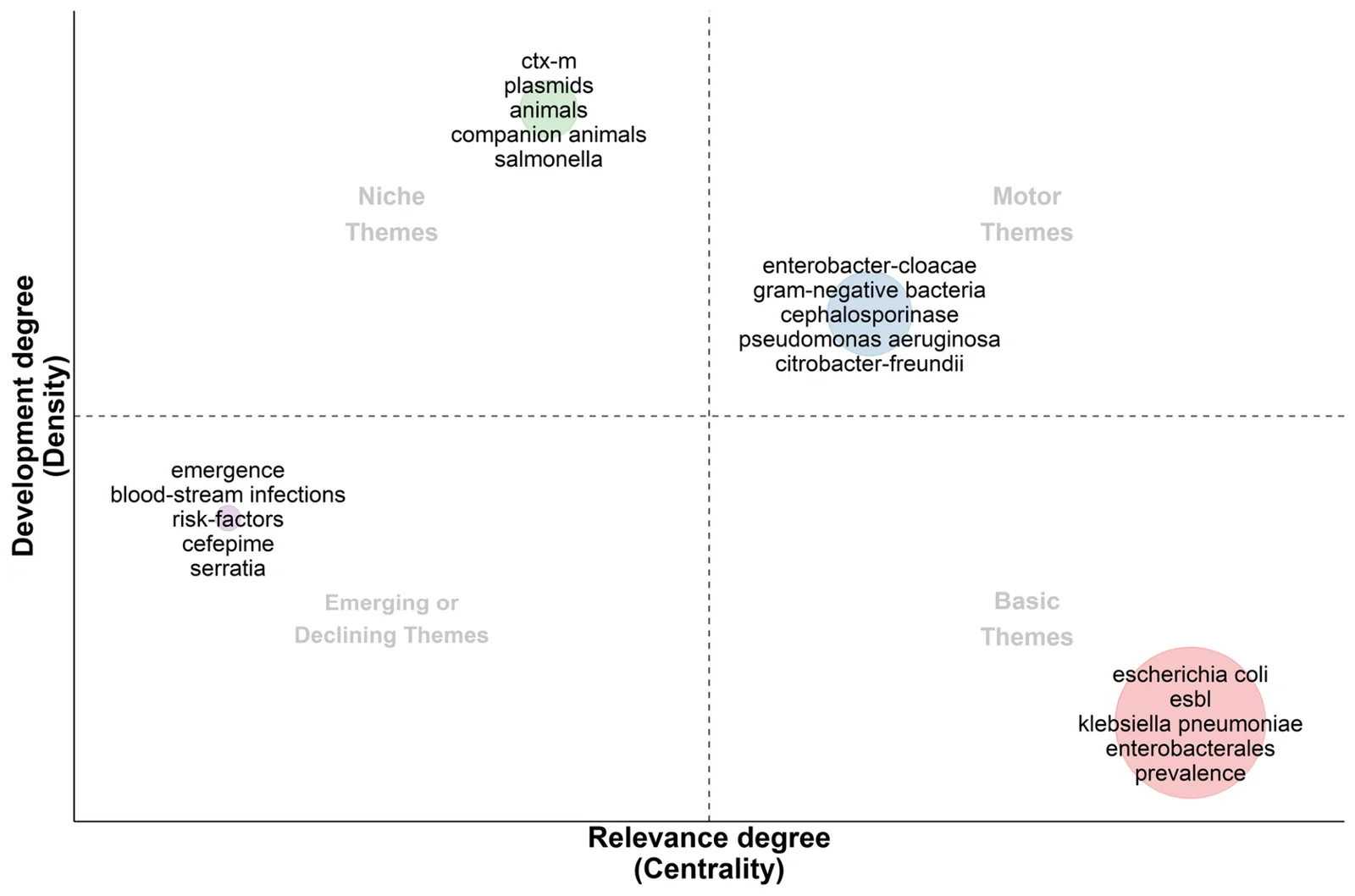
Thematic map of the AmpC β-lactamase literature. Themes were generated from the All Keywords field after synonym standardization and stop-word removal. The map positions thematic clusters according to centrality and density, classifying them as motor themes, basic themes, niche themes, or emerging or declining themes.

The thematic map analysis identified four main clusters. In the Basic Themes quad-187 rant, one cluster comprised “*Escherichia coli*”, “ESBL”, “*Klebsiella pneumoniae*”, “*Enterobacterales*”, and “prevalence”. In the Motor Themes quadrant, “*Enterobacter cloacae”*, “Gram-negative bacteria”, “cephalosporinase”, “*Pseudomonas aeruginosa*”, and “Citrobacter freundii” clustered together. In the Niche Themes quadrant, a separate cluster was formed by “CTX-M”, “plasmids”, “animals”, “companion animals”, and “*Salmonella*”. In the Emerging or Declining Themes quadrant, the cluster included “emergence”, “bloodstream infections”, “risk factors”, “cefepime”, and “*Serratia*”. The thematic map analysis is presented in Figure 12.

In the expanded search sensitivity corpus, three thematic clusters were identified. The cluster comprising *Escherichia coli*, ESBL, *Klebsiella pneumoniae*, *Enterobacterales*, and “prevalence” shifted from the Basic Themes quadrant to the Motor Themes quadrant. The Enterobacter cloacae–“cephalosporinase”–“Gram-negative bacteria”–*Pseudomonas aeruginosa–Citrobacter freundii* cluster was located near the boundary between the Basic and Emerging or Declining Themes quadrants. The former Niche Themes cluster was not identified as a separate cluster. The clinically oriented cluster remained near the boundary between the Niche and Emerging or Declining Themes quadrants, retaining “emergence,” “bloodstream infections,” “risk factors,” and “cefepime,” while “therapy” replaced *Serratia* (Figure S5).

In the stop-word sensitivity analysis, three thematic clusters were identified. The cluster containing *Escherichia coli*, ESBL, and *Enterobacterales* incorporated the reintroduced general terms “antibiotic resistance” and “genes” and was positioned near the boundary between the Motor and Basic Themes quadrants. A second cluster comprising “resistance,” *Enterobacter cloacae*, “emergence,” “bloodstream infections,” and “Gram-negative bacteria” was located in the Niche Themes quadrant. Klebsiella pneumoniae formed a third cluster with “epidemiology,” “infections,” “molecular characterization,” and “plasmid-mediated AmpC” near the boundary between the Emerging or Declining and Basic Themes quadrants. The former CTX-M–plasmid–animal cluster was not retained as a separate cluster (Figure S6).

**Figure 13:**
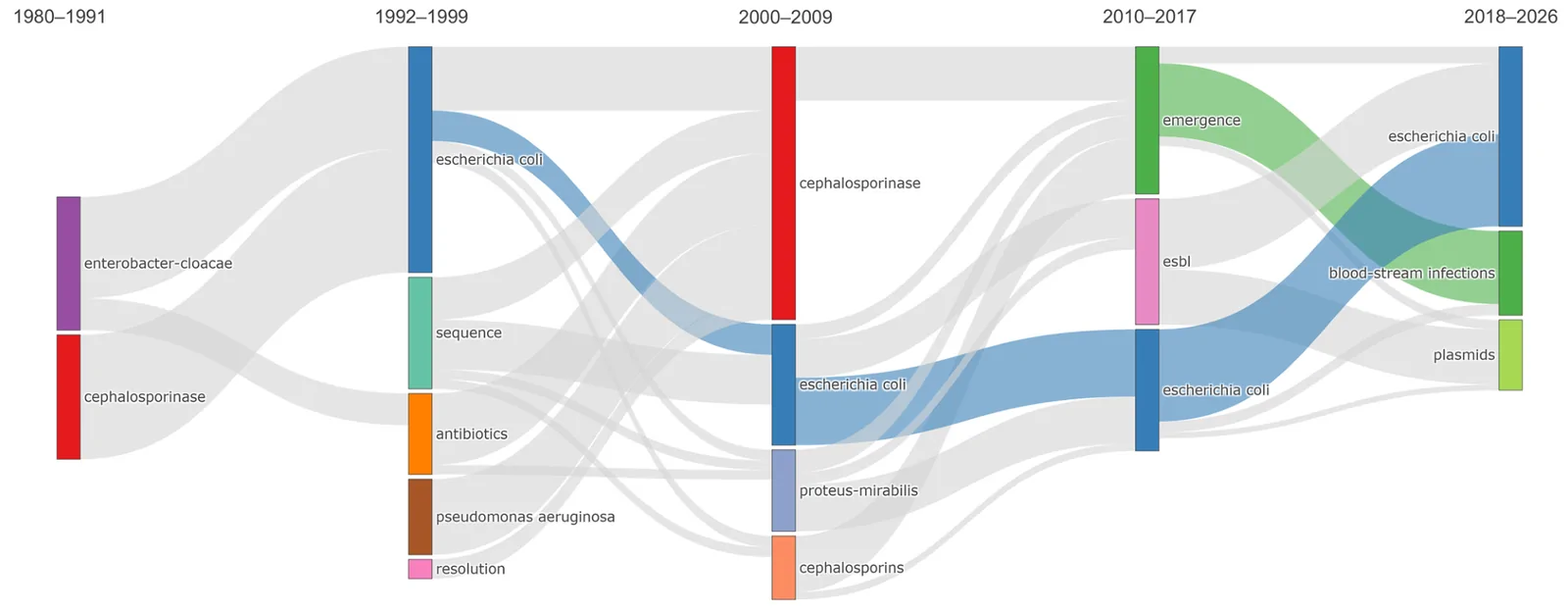
Thematic evolution of AmpC β-lactamase research across five publication periods. The Sankey diagram shows thematic transitions across five publication periods. Node size represents theme frequency, and band width indicates thematic overlap between consecutive periods.

The thematic evolution analysis identified 17 period-specific thematic nodes across five periods. “*Enterobacter cloacae*” and “cephalosporinase” were identified in 1980–1991, while *Escherichia coli* first appeared in 1992–1999 and remained present throughout the subsequent periods. The 2010–2017 period comprised “emergence,” “ESBL,” and *Escherichia coli*, whereas the terminal themes in 2018–2026 were *Escherichia coli*, “bloodstream infections,” and “plasmids” (Figure 13).

In the expanded search sensitivity corpus, the number of thematic nodes increased from 17 to 24. The Escherichia coli pathway was retained and extended to 1980–1991. In 2018–2026, Escherichia coli remained the only terminal theme shared with the primary analysis; “bloodstream infections” and “plasmids” were not retained, whereas *Enterobacter cloacae*, “susceptibility,” and “carbapenemase” appeared as terminal themes (Figure S7).

In the stop-word sensitivity analysis, 24 thematic nodes were identified, and the *Escherichia coli* pathway from 1992 to 1999 onward was retained. The reintroduced terms “genes,” “resistance,” and “antibiotic resistance” appeared in more than one period. Compared with the primary analysis, “bloodstream infections” was identified in 2010–2017 rather than 2018–2026, and “plasmids” was not retained. The terminal themes were *Escherichia coli*, “resistance,” “antibiotic resistance,” and “susceptibility” (Figure S8).

**Figure 14:**
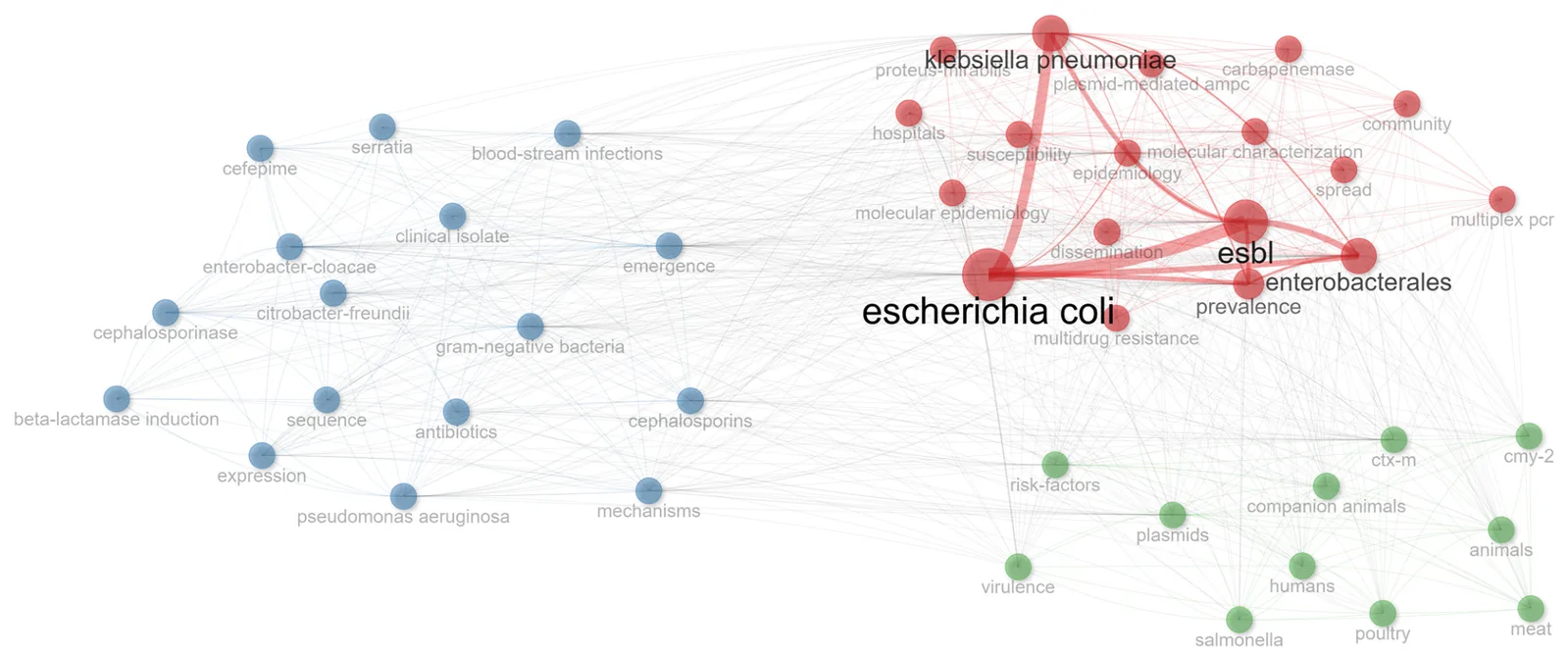
Co-occurrence network of the AmpC β-lactamase literature. The network was generated from the All Keywords field after synonym standardization and stop-word removal. Nodes represent terms, links represent co-occurrence relationships between terms, and node size reflects term weight within the network. Clusters were generated using the Louvain algorithm.

The keyword co-occurrence network comprised three major clusters. The largest and most central cluster was dominated by *Escherichia coli*, ESBL, *Klebsiella pneumoniae*, and *Enterobacterales*, with strong links to prevalence, epidemiology, dissemination, and plasmid-mediated AmpC. A second cluster included chromosomal AmpC-related terms such as *Enterobacter cloacae*, *Citrobacter freundii*, *Pseudomonas aeruginosa*, cephalosporinase, β-lactamase induction, expression, cefepime, and bloodstream infections. The third cluster comprised terms related to animals, poultry, meat, humans, companion animals, plasmids, CTX-M, CMY-2, and virulence (Figure 14).

## 3. Discussion

Using bibliometric methods, this study systematically maps the scientific production dynamics, citation structure, and conceptual evolution of the AmpC β-lactamase literature. To the best of our knowledge, this is the first bibliometric analysis specifically focused on AmpC. The main finding is that the literature is multidimensional, with scientific output increasing over time and research themes receiving varying emphasis across different periods.

According to the annual scientific production curve, AmpC research gained clear momentum from the late 1990s onward. One of the key developments preceding this acceleration was the increasingly detailed characterization of plasmid-mediated AmpC genes and their possible species origins during the 1990s [3,4]. This shift helped reposition AmpC from a mechanism understood primarily in terms of chromosomal class C β-lactamases to an important component of transferable resistance epidemiology. During the same period, reports of resistance development during treatment with third-generation cephalosporins, particularly among *Enterobacter* strains, made the clinical consequences of inducible chromosomal AmpC expression more visible [23,24,25,26]. Taken together, the characterization of plasmid-mediated dissemination and the demonstration of the clinical impact of inducible chromosomal AmpC were temporally associated with the increase in scientific output observed from the 2000s onward. The fact that all of the 10 most productive sources were indexed in SCI-E, that eight were classified as Q1 or Q2 in the 2025 Journal Citation Reports, and that six overlapped with the list of most locally cited sources suggests that AmpC research has remained a sustained topic in highly visible core journals in antimicrobial resistance and clinical microbiology. The close fit to Bradford’s law, with eight sources constituting the core zone, further suggests that AmpC publications were concentrated within a relatively limited group of journals.

The ten most locally cited documents were predominantly review articles, comprising seven reviews and three original research articles. At the top of this list was Jacoby’s review entitled “AmpC β-Lactamases,” which was also prominent in the author-level local citation analysis. Its citation strength may be attributed to its broad coverage of the field’s core themes, including chromosomal and plasmid-mediated AmpC mechanisms, diagnostic approaches, clinical relevance, and therapeutic implications, as well as its publication in *Clinical Microbiology Reviews*, one of the field’s leading journals [3]. The second-ranked publication described multiplex PCR-based detection, whereas the third-ranked publication reviewed plasmid-mediated AmpC β-lactamases. The prominence of these works suggests sustained interest in the epidemiological and clinical distinction between plasmid-mediated and chromosomal AmpC β-lactamases [4,12]. The prominence of several classic reviews outside the analyzed dataset, including foundational works on β-lactamase classification and resistance as well as a major ESBL review, further suggests that the AmpC literature is embedded within the broader β-lactamase field, particularly in relation to extended-spectrum cephalosporin resistance, difficulties in phenotypic differentiation, and the wider epidemiology of Gram-negative resistance [2,20].

The most frequent keywords suggest that the AmpC literature has developed at the intersection of chromosomal AmpC, plasmid-mediated resistance dissemination, ESBL-related β-lactamase epidemiology, and clinical management questions. The prominence of terms such as *Escherichia coli*, ESBL, *Klebsiella pneumoniae*, and *Enterobacterales* indicates that AmpC research has largely been shaped within the *Enterobacterales* context. In addition, the frequent occurrence of terms such as plasmids and plasmid-mediated AmpC suggests that AmpC research has not been limited to natural or inducible chromosomal mechanisms; acquired plasmid-mediated AmpC enzymes have also emerged as a distinct and substantial research focus. This finding is consistent with the increasing clinical and epidemiological importance of plasmid-mediated AmpC enzymes, particularly in species such as *E. coli* and *K. pneumoniae* [27,28].

The high frequencies of ESBL and CTX-M—one of the most globally prevalent ESBL types—together with the prominence of the terms “plasmids” and “plasmid-mediated AmpC,” are among the notable findings of this analysis [29]. The prominence of these terms suggests that AmpC is frequently examined within the same resistance framework as ESBLs. As an illustrative example, plasmid-mediated AmpC genes were concurrently detected in 16.7% of ESBL-producing *Escherichia coli* and *Klebsiella* isolates in one primary study [28]. This close relationship between AmpC and ESBL research is not only epidemiological. Plasmid-mediated AmpC production or high-level AmpC expression may complicate the phenotypic detection of ESBLs, thereby linking these two research areas at the diagnostic and methodological levels as well [30].

Terms representing natural or inducible chromosomal AmpC producers, including *Enterobacter cloacae*, *Pseudomonas aeruginosa*, and *Citrobacter freundii*, were also among the 20 most frequent keywords. Clinically oriented terms such as bloodstream infections, risk factors, and cefepime likewise ranked among the leading terms. This distribution suggests that the literature encompasses both the biological basis of AmpC production and its clinical implications, particularly in invasive infections, where the selection among cefepime, carbapenems, and other β-lactam options remains central to clinical management [7].

The trend topic analysis indicates that molecular characterization and species-focused resistance distribution remained prominent themes in the AmpC literature for an extended period, while concepts related to clinical management and multidrug resistance have become more visible in recent years. Throughout the 2010s, the concurrent visibility of chromosomal AmpC producers, plasmid-mediated AmpC enzymes, and ESBL-related concepts such as CTX-M suggests that AmpC research evolved along interconnected axes of molecular epidemiology, species-specific resistance distribution, and β-lactamase co-occurrence. The increasing prominence of cefepime, bloodstream infections, and risk factors from 2018 onward points to a growing focus on patient-centered clinical questions and treatment-oriented research. This broader clinical orientation was largely retained in the sensitivity analyses, particularly through the recurring presence of bloodstream infections and risk factors, although cefepime and CTX-M were not consistently displayed. This finding is consistent with recent literature suggesting that treatment selection in AmpC-producing *Enterobacterales* infections, clinical outcomes in invasive infections such as bacteremia, and the identification of risk factors have gained increasing attention [31,32,33,34,35,36]. The presence of multidrug resistance, carbapenemase, and virulence among the most recent terms further suggests that AmpC research is increasingly being discussed within a broader antimicrobial resistance context, including the co-carriage of resistance genes and pathogen virulence. Multidrug resistance was retained across all sensitivity analyses, whereas carbapenemase and virulence were not displayed in the author-keyword-only analysis; the appearance of general terms such as resistance and antibiotic resistance after stop-word reintroduction also indicates some dependence on preprocessing choices. As an illustration of this emerging research direction, a recent experimental study reported that some acquired AmpC β-lactamases may not only confer β-lactam resistance but also affect bacterial fitness, motility, and pathogenicity [37].

The keyword co-occurrence network suggests that the AmpC literature is organized around three interconnected conceptual axes. The first and most central axis is centered on *Escherichia coli*, ESBL, *Klebsiella pneumoniae*, and *Enterobacterales* and integrates plasmid-mediated AmpC, molecular epidemiology, prevalence, dissemination, diagnostic characterization, and multidrug resistance. The second axis represents the classical biological and clinical dimensions of chromosomal or inducible AmpC production, linking *Enterobacter cloacae*, *Citrobacter freundii*, *Pseudomonas aeruginosa*, cephalosporinase induction, expression, and resistance mechanisms with cefepime and bloodstream infections. The third axis comprises terms related to animals, poultry, meat, humans, companion animals, *Salmonella*, plasmids, CTX-M, and CMY-2 and may therefore be interpreted as a One Health-oriented research line connecting animal reservoirs, the food chain, and human health. Although co-occurrence relationships do not themselves demonstrate transmission, studies documenting ESBL/AmpC-producing *E. coli* across poultry production systems and associated environmental sources support the broader interpretation that plasmid-mediated β-lactamases form part of a resistance ecology extending beyond clinical settings [38,39,40].

The thematic map analysis complements the network structure by distinguishing broadly relevant themes from more developed, specialized, or potentially emerging research areas. The cluster centered on *Escherichia coli*, ESBL, *Klebsiella pneumoniae*, *Enterobacterales*, and prevalence occupied the basic-themes quadrant, indicating high relevance to the overall field but comparatively lower internal development. In contrast, the cluster comprising chromosomal or inducible AmpC producers, including *Enterobacter cloacae*, *Pseudomonas aeruginosa*, and *Citrobacter freundii*, was positioned among the motor themes, supporting the interpretation that classical AmpC biology constitutes a well-developed and central research domain. In the primary thematic map, concepts related to animal reservoirs, the food chain, plasmids, and related resistance determinants formed a specialized One Health-oriented cluster. However, the absence of this distinct cluster in the thematic-map sensitivity analyses indicates that its boundaries and degree of specialization were dependent on corpus composition and preprocessing choices. Clinical concepts such as cefepime, bloodstream infections, and risk factors were positioned in the emerging-or-declining quadrant. Although the exact quadrant position of this clinically oriented cluster varied across the sensitivity analyses, it remained near the boundary between the Niche Themes and Emerging or Declining Themes quadrants. Moreover, the recurrent presence of bloodstream infections and risk factors, together with their recent temporal visibility, is consistent with their interpretation as a developing clinical research direction.

The thematic evolution analysis adds a temporal dimension to these cross-sectional findings. The earliest periods were dominated by Enterobacter cloacae, cephalosporinase, sequence, and Pseudomonas aeruginosa, reflecting an initial emphasis on chromosomal AmpC biology, enzymology, and molecular characterization. From 2000 onward, *Escherichia coli* became a persistent thematic bridge, while species- and cephalosporinase-centered themes increasingly connected with ESBL and emergence. In the primary analysis, the most recent period included *E. coli*, bloodstream infections, and plasmids, indicating expansion toward clinical questions and transferable resistance. Although the exact terminal themes varied, the sensitivity analyses showed a comparable broadening: susceptibility and carbapenemase emerged in the expanded-search analysis, whereas resistance and antibiotic resistance appeared after the reintroduction of general terms. Thus, the evolution analyses collectively support a transition from an early focus on chromosomal AmpC biology toward a broader and more complex antimicrobial resistance ecology encompassing plasmid-mediated dissemination, clinical infections, therapeutic susceptibility, and associated resistance mechanisms.

The targeted manual classification further highlighted a distinct structural and enzymological research axis encompassing AmpC enzymology, protein structure, catalytic mechanisms, inhibitor discovery, and computational screening. Although less prominent than the epidemiological and clinical domains, this axis represents an identifiable mechanistic component of the broader AmpC literature and reflects the continuing relevance of structure- and mechanism-based approaches to understanding AmpC function and developing potential inhibitors.

The leading position of the United States in both publication output by corresponding author country and total citation count suggests that country-level visibility in the AmpC literature may be related not only to regional resistance burden but also to research infrastructure, publication capacity, and representation in high-visibility journals. The high publication volume from India and China also appears consistent with their large populations, substantial Gram-negative resistance burden, and expanding antimicrobial resistance research capacity. Indeed, global antimicrobial resistance (AMR) burden studies suggest that AMR will remain a major public health challenge in highly populated regions, particularly South Asia [1].

The predominance of single-country publications over multiple-country publications suggests that international collaboration has remained relatively limited in the AmpC literature. The country collaboration network further indicated that collaborative ties were concentrated around a small number of highly connected countries, particularly the United States, Spain, and France, whereas many other countries occupied relatively peripheral positions. This pattern may partly reflect differences in research infrastructure, access to international networks, and capacity for multicenter studies. Given the dissemination of plasmid-mediated AmpC enzymes, clonal expansion, and resistance transmission along the food–animal–human interface, these findings are consistent with the need to strengthen multicenter and cross-border surveillance networks [13].

## 4. Materials and Methods

### 4.1. Study Design and Data Source

This study was designed as a descriptive bibliometric analysis to evaluate scientific production trends, authors, influential journals and publications, country-level contributions, citation structure, and thematic development in the AmpC β-lactamase literature. The Web of Science Core Collection was used as the data source. The search was restricted to publications indexed in the Science Citation Index Expanded (SCI-E) and Emerging Sources Citation Index (ESCI).

### 4.2. Search Strategy and Study Selection

The search strategy was structured in a stepwise manner to capture the AmpC β-lactamase literature as specifically as possible while excluding irrelevant records. In the first step, a search using only the term “AmpC” in the title field retrieved 1438 records. To reduce heterogeneity in publication types, document types were restricted to original articles (“article”) and review articles (“review article”), which reduced the number of records to 1233. Subsequently, only studies published in English were included, resulting in a dataset of 1201 publications.

During the review of the search strategy, it was observed that the term “AmpC” could also be used as an abbreviation for various concepts in scientific fields outside microbiology. Therefore, an additional topic-field filter was applied to exclude false-positive records and increase the subject specificity of the dataset. In the final step, among publications containing “AmpC” in the title field, only records that included microbiological terminology related to β-lactamases, antimicrobial resistance, *Enterobacterales*-associated bacteria, and AmpC-related resistance mechanisms in the topic field were selected.

The final search strategy was defined as follows:

TI = (AmpC) AND TS = (beta-lactamase OR beta-lactamases OR beta lactamase OR beta lactamases OR β-lactamase OR β-lactamases OR lactamase OR lactamases OR resistance OR Enterobacter OR Enterobacterales OR Enterobacteriaceae OR Klebsiella OR Citrobacter OR Escherichia OR carbapenem OR carbapenems OR cefepime).

This filtering process yielded a total of 1183 records. No lower publication-year limit was applied, and the final dataset covered publications from 1980 to 2026. The 18 records excluded after the application of the additional topic-field filter were manually screened using their titles, abstracts, and available bibliographic metadata. No clinically or microbiologically relevant AmpC β-lactamase publications were identified among the excluded records.

To complement this exclusion audit with an assessment of benchmark recall, an outcome-neutral benchmark seed set comprising 15 established publications was constructed before examining the retrieval status of the individual records. The seed set covered three predefined research axes: foundational and diagnostic research, clinical and therapeutic research, and One Health and food-chain research. Each seed publication was individually assessed for retrieval by the complete final search strategy. Overall benchmark recall rate was calculated as the proportion of seed publications retrieved by the search, and axis-stratified recall was calculated separately for each of the three research axes. Consequently, a total of 1183 publications were included in the final bibliometric analyses.

### 4.3. Data Preparation and Bibliometric Analysis

The search strategy was applied to the Web of Science Core Collection on 15 June 2026, and the retrieved records were exported on the same day as “Full Record and Cited References” in .txt format. Because the records were exported in three separate files, these files were imported and merged in R before analysis. Bibliometric analyses and visualizations were performed using R software version 4.6.0, the bibliometrix package version 5.4.0, and the Biblioshiny interface [11,41].

Annual and Cumulative Scientific Production analyses were used to evaluate annual and cumulative trends in scientific output. Most Relevant Sources and Most Local Cited Sources analyses were performed to identify the most productive and locally influential journals, while Bradford’s Law was applied to determine the core journals in the field. For the ten most productive sources, indexing status was verified using the Clarivate Master Journal List. Journal Impact Factor and category-specific quartile data were obtained from the 2026 edition of Journal Citation Reports, based on 2025 JIF data. Most Relevant Authors, Most Local Cited Authors, and Authors’ Local Impact by h-index analyses were used to assess author productivity and influence. Corresponding Author’s Countries and Most Cited Countries analyses were conducted to examine country-level scientific production and citation impact, while the Country Collaboration Network analysis was used to characterize international research collaboration. Most Local Cited Documents and Most Local Cited References analyses were used to describe the citation structure of the AmpC literature. Most Relevant Words, Co-occurrence Network, and Thematic Map analyses were performed to characterize the conceptual structure and major research themes. Thematic Evolution analysis was used to examine the development, continuity, merging, and divergence of research themes across predefined time periods, while Trend Topics analysis was used to evaluate temporal shifts in the prominence of individual terms.

In addition to the standard bibliometric analyses, the structural and enzymological component of the corpus was assessed through a targeted manual classification. Web of Science research areas and categories potentially related to biochemistry, molecular biology, biophysics, crystallography, medicinal chemistry, and computational research were reviewed. This process identified 90 candidate publications. As an author-based cross-check, all 15 publications authored by Shoichet BK within the final corpus were also reviewed. Fourteen of these publications were already included in the category-based candidate set, whereas one additional publication was identified, resulting in 91 unique candidate publications. The titles and abstracts of these publications were manually screened. Publications were classified within the structural and enzymological research axis when their primary focus was AmpC enzymology, protein structure, inhibitor design, molecular docking, or virtual screening. Publications primarily addressing clinical isolates, epidemiology, diagnostic methods, gene regulation, or antimicrobial susceptibility were not classified within this axis.

### 4.4. Expanded Search Sensitivity Analysis

To assess whether AmpC-focused publications without the term “AmpC” in their titles may have been missed, a targeted sensitivity search was conducted in the Web of Science Core Collection on 22 July 2026 using the query TS = (AmpC) AND TI = (Citrobacter OR Enterobacter OR “Klebsiella aerogenes” OR CMY OR DHA OR FOX OR ACT OR MIR OR MOX OR ACC) NOT TI = (AmpC). The primary corpus was retained because the study was designed to provide a high-specificity bibliometric characterization of publications explicitly framed around AmpC at the title level. The additional 310 records identified through this search were therefore merged with the primary corpus to construct an expanded sensitivity corpus of 1493 publications, rather than being incorporated into the primary analysis. The same database coverage, document-type and language restrictions, preprocessing procedures, thesaurus and stop-word files, field selection, frequency thresholds, and analytical parameters were applied to both corpora. The Most Relevant Words, Trend Topics, Thematic Map, and Thematic Evolution analyses were then repeated in the expanded corpus.

### 4.5. Keyword and Conceptual Structure Analysis

The “All Keywords” field was used for keyword and conceptual structure analyses. The availability of author keywords and Keywords Plus terms was evaluated across four publication periods: 1980–1999, 2000–2009, 2010–2017, and 2018–2026. For each period, we calculated the proportions of documents containing at least one author keyword, at least one Keywords Plus term, and at least one term from either field. Because keyword coverage was substantially lower before 2000, the trending-topics analysis was restricted to publications from 2000 to 2026. An additional sensitivity analysis using author keywords only was performed with the same frequency criteria as the primary All Keywords analysis.

Before analysis, spelling differences, hyphenation patterns, singular–plural variants, abbreviations, character-encoding variants, and terms referring to the same concept in the AmpC literature were standardized using a synonym dictionary. Taxonomic terms were harmonized using a corpus-informed, context-sensitive approach. Genus-level terms were merged with a species or species-complex label only when their use within the corpus predominantly referred to that taxonomic concept. Accordingly, “*Citrobacter*” was harmonized under “*Citrobacter freundii*” and “*Enterobacter*” under “*Enterobacter cloacae*” In contrast, “*Klebsiella*” was retained as a separate genus-level term because its use could encompass multiple clinically relevant species, including *Klebsiella pneumoniae* and *Klebsiella aerogenes*.

The stop-word list was defined according to corpus specificity and thematic discriminatory value. The corpus-defining term “AmpC” and highly general terms used across heterogeneous research contexts, including “resistance,” “antimicrobial resistance,” “infection/infections,” and “gene/genes,” were treated as stop-words in the primary analyses because they provided limited discrimination among specific thematic domains. More specific compound terms denoting distinct resistance phenotypes or clinical syndromes, such as “multidrug resistance” and “bloodstream infections,” were retained. However, because “resistance” and “infection/infections” are lexical components of these retained compound terms, their exclusion as standalone stop-words could potentially increase the relative prominence of “multidrug resistance” and “bloodstream infections” in the keyword-based analyses. To assess this potential specification-related bias, a stop-word sensitivity analysis was performed in which “resistance,” harmonized variants of “antimicrobial resistance,” “infection/infections,” and “gene/genes” were reintroduced into the analysis. Trend Topics, Thematic Map, and Thematic Evolution analyses were then repeated using the same dataset, synonym dictionary, frequency thresholds, and all other analytical parameters as in the primary analyses.

The synonym dictionary, stop-word list, and main parameters used for the Co-occurrence Network, Thematic Map, Trend Topics, and Thematic Evolution analyses are provided in the Supplementary Methods section of the Supplementary Materials.

## 5. Conclusions

This bibliometric analysis suggest that the AmpC β-lactamase literature has broadened from an earlier emphasis on chromosomal class C β-lactamase biology toward a multidimensional research area encompassing plasmid-mediated dissemination, resistance ecology intersecting with ESBLs and carbapenemases, a cluster interpretable as a One Health axis, a distinct structural and enzymological research axis and clinical treatment questions. The increasing visibility of multidrug resistance, carbapenemase, and virulence in recent years further suggests that AmpC research is increasingly situated within a broader antimicrobial resistance context and may reflect growing attention to the co-dissemination of resistance determinants and pathogen virulence traits.

## 6. Limitations

This study has several limitations. First, the search strategy required the term “AmpC” to appear in the title field. Although this approach increased topic specificity, it may have missed some publications in which AmpC was addressed as a secondary resistance mechanism, a subgroup variable, or a component of a broader clinical or molecular context. In addition, studies focusing on specific AmpC-family enzymes or variants, such as CMY, DHA, FOX, or MIR, may not have been captured if the term “AmpC” was not explicitly included in the title. Second, because bibliometric analyses are based on quantitative indicators such as publication counts, citation counts, keyword frequency, and co-occurrence patterns, these findings do not directly reflect the methodological quality, level of evidence, or clinical applicability of the included studies. Third, keyword-based analyses depend on which terms are retrieved from the All Keywords field, how these terms are indexed in the database, and how they are standardized before analysis. Although the sensitivity analyses generally supported the broader conceptual patterns, the ranking of individual terms, cluster composition, quadrant placement, and thematic evolution pathways varied to some extent according to corpus definition and preprocessing choices. The author-keyword-only analysis was also more informative for recent periods because author-keyword coverage was limited in earlier records. Therefore, the findings from the trend topic analysis, co-occurrence network, thematic map and thematic evolution analysis should be interpreted not as definitive classifications, but as exploratory indicators of broader research patterns in the AmpC literature. As such, the findings of this study should be viewed as a bibliometric mapping of the structure and conceptual evolution of the AmpC literature, rather than as a systematic review directly assessing the clinical evidence level or methodological quality of the included studies.

## Data Availability

The bibliographic data used in this study were retrieved from the Web of Science Core Collection. The search strategy, preprocessing procedures, and analysis parameters are described in the Methods section and Supplementary Materials. The processed data supporting the findings of this study are available from the corresponding author upon reasonable request, subject to Web of Science database licensing restrictions.

## Supplementary Materials

The following supporting information can be downloaded at: https://www.mdpi.com/article/10.3390/antibiotics15080777/s1. Table S1: Coverage of the Predefined Benchmark Seed Set by the Final Search Strategy Across Research Axes; Table S2: Publications Classified Within the Structural and Enzymological Research Axis After Targeted Manual Screening; Table S3: General characteristics of the bibliometric dataset; Table S4: Missing data analysis of the bibliometric dataset; Table S5: Keyword-field coverage according to publication period; Table S6: Web of Science Indexing and 2025 JIF Characteristics of the Ten Most Relevant Sources; Figure S1: Most relevant keywords in the expanded-search sensitivity analysis. Figure S2: Trend topics in the expanded-search sensitivity analysis. Figure S3: Trend topics in the stop-word sensitivity analysis. Figure S4: Trend topics in the author-keyword-only sensitivity analysis. Figure S5: Thematic map in the expanded-search sensitivity analysis. Figure S6: Thematic map in the stop-word sensitivity analysis. Figure S7: Thematic evolution in the expanded-search sensitivity analysis. Figure S8: Thematic evolution in the stop-word sensitivity analysis. Trend Topics Analysis Parameters; Thematic map and Thematic Evolution Analysis Parameters; Co-occurrence Network Analysis Parameters; Synonyms List; Stop-Words List. References [2,3,4,7,12,13,31,32,33,34,35,36,38,39,40] are cited in Table S1 of the Supplementary Materials.
