# Supplementary material for: Mapping the Thematic Structure and Evolution of AmpC β-Lactamase Research: A Bibliometric Analysis
Source: Antibiotics (Basel). 2026 Aug 13;15(8):777. doi: 10.3390/antibiotics15080777 (PMC13509352; doi:10.3390/antibiotics15080777)
Supplement: Supplementary file 1 [file antibiotics-15-00777-s001.zip › antibiotics-4429452-supplementary.pdf]

Table S1. Coverage of the Predefined Benchmark Seed Set by the Final Search Strategy Across Research Axes

Research axis	Seed publication	Publication scope	Retrieved in final search
Foundational and diagnostic	Bush K, Jacoby GA. *Updated functional classification of beta-lactamases.* Antimicrob Agents Chemother. 2010;54:969–976.	Broad functional classification of β-lactamases	No
Foundational and diagnostic	Jacoby GA. *AmpC beta-lactamases.* Clin Microbiol Rev. 2009;22:161–182.	Foundational review of AmpC β-lactamases	Yes
Foundational and diagnostic	Philippon A, Arlet G, Jacoby GA. *Plasmid-determined AmpC-type beta-lactamases.* Antimicrob Agents Chemother. 2002;46:1–11.	Foundational review of plasmid-mediated AmpC β-lactamases	Yes
Foundational and diagnostic	Pérez-Pérez FJ, Hanson ND. *Detection of plasmid-mediated AmpC beta-lactamase genes in clinical isolates by using multiplex PCR.* J Clin Microbiol. 2002;40:2153–2162.	Molecular detection and diagnostic methodology	Yes
Foundational and diagnostic	Thomson KS. *Extended-spectrum-beta-lactamase, AmpC, and carbapenemase issues.* J Clin Microbiol. 2010;48:1019–1025.	Diagnostic and interpretative review of major β-lactamase mechanisms	Yes
Clinical and therapeutic	Tamma PD, Heil EL, Justo JA, et al. *Infectious Diseases Society of America 2024 guidance on the treatment of antimicrobial-resistant Gram-negative infections.* Clin Infect Dis. 2024:ciae403.	General clinical guidance on antimicrobial-resistant Gram-negative infections	No
Clinical and therapeutic	Shoji T, Otsubo Y, Horikoshi Y. *Definitive cefepime versus carbapenems for bacteremia caused by Enterobacterales with a risk of chromosomal AmpC production in children.* J Infect Chemother. 2026;32:102923.	Comparative effectiveness study of cefepime and carbapenems	Yes
Clinical and therapeutic	Tebano G, Campoli C, Meschiari M, et al. *Exploring heterogeneity in antibiotic choices for bloodstream infections caused by AmpC-producing Enterobacterales: results from a case-vignette study.* Eur J Clin Microbiol Infect Dis. 2026;45:125–133.	Clinical treatment-decision study	Yes
Clinical and therapeutic	Mitsuboshi S, Inose R, Yanagisawa R, et al. *Efficacy and safety of various antibiotics in bacteremia caused by AmpC-producing Enterobacteriaceae, comparing carbapenems: a systematic review and network meta-analysis.* Biol Pharm Bull. 2026;49:414–421.	Systematic review and network meta-analysis of therapeutic alternatives	Yes
Clinical and therapeutic	Saleh KB, Alowais SA, Aldairem AO, et al. *Comparative efficacy and safety of carbapenems vs noncarbapenem antibiotics in AmpC-producing Enterobacterales infections: a systematic review and meta-analysis.* Int J Infect Dis. 2026;167:108563.	Systematic review and meta-analysis of carbapenem and non-carbapenem treatment	Yes
Clinical and therapeutic	Onorato L, de Luca I, Salvati A, et al. *Piperacillin/tazobactam vs. cefepime or carbapenems for the treatment of bloodstream infections due to bacteria producing chromosomal AmpC beta-lactamase: a systematic review and meta-analysis.* Infection. 2025;53:1141–1153.	Systematic review and meta-analysis of bloodstream-infection treatment	Yes
One Health and food-chain	Huijbers PMC, Graat EAM, van Hoek AHAM, et al. *Transmission dynamics of extended-spectrum β-lactamase and AmpC β-lactamase-producing Escherichia coli in a broiler flock without antibiotic use.* Prev Vet Med. 2016;131:12–19.	Transmission of ESBL/AmpC-producing *E. coli* in poultry production	Yes
One Health and food-chain	Laube H, Friese A, von Salviati C, et al. *Transmission of ESBL/AmpC-producing Escherichia coli from broiler chicken farms to surrounding areas.* Vet Microbiol. 2014;172:519–527.	Environmental transmission from poultry farms	Yes
One Health and food-chain	Casella T, Nogueira MCL, Saras E, et al. *High prevalence of ESBLs in retail chicken meat despite reduced use of antimicrobials in chicken production, France.* Int J Food Microbiol. 2017;257:271–275.	Food-chain antimicrobial-resistance study including AmpC-producing isolates	No
One Health and food-chain	Ewers C, Bethe A, Semmler T, et al. *Extended-spectrum β-lactamase-producing and AmpC-producing Escherichia coli from livestock and companion animals, and their putative impact on public health: a global perspective.* Clin Microbiol Infect. 2012;18:646–655.	Global One Health review of animal reservoirs and public-health implications	Yes

The benchmark seed set was predefined to represent three research axes: foundational and diagnostic, clinical and therapeutic, and One Health and food-chain research. Publication scope was assigned according to the primary focus of each study. "Yes" indicates that the publication was identified by the final search strategy and retained in the final dataset after application of the eligibility criteria; "No" indicates that it was not retrieved. Overall recall was 80.0% (12/15), with axis-specific recall of 80.0% (4/5), 83.3% (5/6), and 75.0% (3/4), respectively. ESBL, extended-spectrum β-lactamase.

Table S2. Publications Classified Within the Structural and Enzymological Research Axis After Targeted Manual Screening

Title	Primary structural/enzymological subaxis	Screening source	DOI / persistent link
Sequence of the cıtrobacter-freundıı os60 chromosomal ampc beta-lactamase gene	Protein sequence / structural characterization	WoS category-based candidate set	10.1111/j.1432-1033.1986.tb09601.x
Sequence and comparatıve-analysıs of 3 enterobacter-cloacae-ampc beta-lactamase genes and theır products	Enzymology / functional characterization	WoS category-based candidate set	10.1042/bj2500753
The role of tyrosıne-150 ın catalysıs of beta-lactam hydrolysıs by ampc beta-lactamase from escherıchıa-colı ınvestıgated by sıte-dırected mutagenesıs	Enzymology / functional characterization	WoS category-based candidate set	10.1021/bi00194a024
Role of asparagıne-152 ın catalysıs of beta-lactam hydrolysıs by escherıchıa-colı ampc beta-lactamase studıed by sıte-dırected mutagenesıs	Enzymology / functional characterization	WoS category-based candidate set	10.1021/bi00023a023
Structural and functional implications of sequence diversity of Pseudomonas aeruginosa genes oriC, ampC and fliC	Enzymology / functional characterization	WoS category-based candidate set	10.1002/elps.1150190414
Structure-based enhancement of boronic acid-based inhibitors of AmpC β-lactamase	Inhibitor discovery / biochemical screening	WoS category-based candidate set; Shoichet BK author-based cross-check	10.1021/jm980343w
Three-dimensional structure of AmpC β-lactamase from Escherichia coli bound to a transition-state analogue: Possible implications for the oxyanion hypothesis and for inhibitor design	Protein structure / catalytic mechanism	WoS category-based candidate set; Shoichet BK author-based cross-check	10.1021/bi981210f
Functional analyses of AmpC β-lactamase through differential stability	Enzymology / functional characterization	WoS category-based candidate set; Shoichet BK author-based cross-check	10.1110/ps.8.9.1816
The complexed structure and antimicrobial activity of a non-β-lactam inhibitor of AmpC β-lactamase	Inhibitor discovery / biochemical screening	WoS category-based candidate set; Shoichet BK author-based cross-check	10.1110/ps.8.11.2330

Title	Primary structural/enzymological subaxis	Screening source	DOI / persistent link
Crystal structures of substrate and inhibitor complexes with AmpC β-lactamase: Possible implications for substrate-assisted catalysis	Protein structure / catalytic mechanism	WoS category-based candidate set; Shoichet BK author-based cross-check	10.1021/ja001676x
Inhibition of AmpC β-lactamase through a destabilizing interaction in the active site	Protein structure / catalytic mechanism	WoS category-based candidate set; Shoichet BK author-based cross-check	10.1021/bi010641m
Structure-based design and in-parallel synthesis of inhibitors of AmpC β-lactamase	Inhibitor discovery / biochemical screening	WoS category-based candidate set; Shoichet BK author-based cross-check	10.1016/S1074-5521(01)00034-5
Structures of ceftazidime and its transition-state analogue in complex with AmpC β-lactamase:: Implications for resistance mutations and inhibitor design	Protein structure / catalytic mechanism	WoS category-based candidate set; Shoichet BK author-based cross-check	10.1021/bi0109358
Structural milestones in the reaction pathway of an amide hydrolase:: Substrate, acyl, and product complexes of cephalothin with AmpC β-lactamase	Protein structure / catalytic mechanism	WoS category-based candidate set; Shoichet BK author-based cross-check	10.1016/S0969-2126(02)00725-6
Structure-based approach for binding site identification on AmpC β-lactamase	Protein structure / catalytic mechanism	WoS category-based candidate set; Shoichet BK author-based cross-check	10.1021/jm020002p
Structure-based discovery of a novel, noncovalent inhibitor of AmpC β-lactamase	Docking / virtual screening / computational modeling	WoS category-based candidate set; Shoichet BK author-based cross-check	10.1016/S0969-2126(02)00799-2
Nanomolar inhibitors of AmpC β-lactamase	Inhibitor discovery / biochemical screening	WoS category-based candidate set; Shoichet BK author-based cross-check	10.1021/ja0288338
Aza-boronic acids as non-β-lactam inhibitors of AmpC-β-lactamase	Inhibitor discovery / biochemical screening	WoS category-based candidate set	10.1016/j.bmcl.2004.05.054
Identification and characteristic analysis of the ampC gene encoding β-lactamase from Vibrio fischeri	Protein sequence / structural characterization	WoS category-based candidate set	10.1016/j.bbrc.2003.12.171
Characteristic analysis of the ampC gene encoding β-lactamase from Photobacterium phosphoreum	Protein sequence / structural characterization	WoS category-based candidate set	10.1016/j.bbrc.2004.11.065
The deacylation mechanism of AmpC β-lactamase at ultrahigh resolution	Protein structure / catalytic mechanism	WoS category-based candidate set; Shoichet BK author-based cross-check	10.1021/ja056806m
Overexpression of the recombinant Enterobacter cloacae P99 AmpC β-lactamase and its mutants based on a φ105 prophage system in Bacillus subtilis	Enzymology / functional characterization	WoS category-based candidate set	10.1016/j.pep.2007.06.001
Differentiation of AmpC beta-lactamase binders vs. decoys using classification kNN QSAR modeling and application of the QSAR classifier to virtual screening	Docking / virtual screening / computational modeling	WoS category-based candidate set	10.1007/s10822-008-9199-2
Structural requirements for the stability of novel cephalosporins to AmpC β-lactamase based on 3D-structure	Protein structure / catalytic mechanism	WoS category-based candidate set	10.1016/j.bmc.2007.11.074
Docking for fragment inhibitors of AmpC β-lactamase	Docking / virtual screening / computational modeling	Shoichet BK author-based cross-check only	10.1073/pnas.0813029106
Structure of AmpC β-lactamase (AmpCD) from an Escherichia coli clinical isolate with a tripeptide deletion (Gly286-Ser287-Asp288) in the H10 helix	Protein structure / catalytic mechanism	WoS category-based candidate set	10.1107/S1744309109014249
Structural study of phenyl boronic acid derivatives as AmpC β-lactamase inhibitors	Protein structure / catalytic mechanism	WoS category-based candidate set; Shoichet BK author-based cross-check	10.1016/j.bmcl.2010.04.007
Virtual screening of AmpC/β-lactamase as target for antimicrobial resistance in Pseudomonas aeruginosa	Docking / virtual screening / computational modeling	WoS category-based candidate set	10.6026/97320630004290
Exploring sequence requirements for C3/C4 carboxylate recognition in the Pseudomonas aeruginosa cephalosporinase: Insights into plasticity of the AmpC β-lactamase	Enzymology / functional characterization	WoS category-based candidate set	10.1002/pro.612
Validation of the AmpC β-Lactamase Binding Site and Identification of Inhibitors with Novel Scaffolds	Docking / virtual screening / computational modeling	WoS category-based candidate set	10.1021/ci300068m
Kinetic Characterization of Fragment Binding in AmpC β-Lactamase by High-Throughput Molecular Simulations	Docking / virtual screening / computational modeling	WoS category-based candidate set	10.1021/ci4006063
Structure-based efforts to optimize a non-β-lactam inhibitor of AmpC β-lactamase	Inhibitor discovery / biochemical screening	WoS category-based candidate set	10.1016/j.bmc.2014.04.051
A fluorescein-labeled AmpC β-lactamase allows rapid characterization of β-lactamase inhibitors by real-time fluorescence monitoring of the β-lactamase-inhibitor interactions	Inhibitor discovery / biochemical screening	WoS category-based candidate set	10.1002/biot.201400861
Comparative Phosphoproteomics Reveals the Role of AmpC β-lactamase Phosphorylation in the Clinical Imipenem-resistant Strain Acinetobacter baumannii SK17	Enzymology / functional characterization	WoS category-based candidate set	10.1074/mcp.M115.051052
Structural basis for the extended substrate spectrum of AmpC BER and structure-guided discovery of the inhibition activity of citrate against the class C β-lactamases AmpC BER and CMY-10	Protein structure / catalytic mechanism	WoS category-based candidate set	10.1107/S2059798316011311
Design, synthesis and biological evaluation of non-covalent AmpC β-lactamases inhibitors	Inhibitor discovery / biochemical screening	WoS category-based candidate set	10.1007/s00044-017-1809-x
Antimicrobial and KPC/AmpC inhibitory activity of functionalized benzosiloxaboroles	Inhibitor discovery / biochemical screening	WoS category-based candidate set	10.1016/j.ejmech.2019.03.028
Studies on the inhibition of AmpC and other β-lactamases by cyclic boronates	Inhibitor discovery / biochemical screening	WoS category-based candidate set	10.1016/j.bbagen.2019.02.004
A novel fluorescent probe for the detection of AmpC beta-lactamase and the application in screening beta-lactamase inhibitors	Inhibitor discovery / biochemical screening	WoS category-based candidate set	10.1016/J.saa.2020.118257

Title	Primary structural/enzymological subaxis	Screening source	DOI / persistent link
Bicyclic Boronates as Potent Inhibitors of AmpC, the Class C β-Lactamase from Escherichia coli	Inhibitor discovery / biochemical screening	WoS category-based candidate set	10.3390/biom10060899
Crystal structure of AmpC BER and molecular docking lead to the discovery of broad inhibition activities of halisulfates against β-lactamases	Docking / virtual screening / computational modeling	WoS category-based candidate set	10.1016/j.csbj.2020.12.015
AmpC Inhibition: An Explicit Approach against Multi-drug Resistance (MDR)	Inhibitor discovery / biochemical screening	WoS category-based candidate set	10.2174/1568026623666230504095005
Aromatic Diboronic Acids as Effective KPC/AmpC Inhibitors	Inhibitor discovery / biochemical screening	WoS category-based candidate set	10.3390/molecules28217362
Machine Learning and Deep Learning Models for Predicting Noncovalent Inhibitors of AmpC β-Lactamase	Docking / virtual screening / computational modeling	WoS category-based candidate set	10.1021/acsomega.4c03834
Insights into the Enhanced Ceftazidime Hydrolysis by Ent385 AmpC β-Lactamase from Multiscale Simulations	Docking / virtual screening / computational modeling	WoS category-based candidate set	10.1021/acscatal.5c02383
In silico targeting of AmpC beta-lactamases in Enterobacter cloacae: unveiling Piperenol B as a potent antimicrobial lead	Docking / virtual screening / computational modeling	WoS category-based candidate set	10.1080/07391102.2024.2446661

Candidate publications were identified through selected Web of Science research areas and categories (n = 90) and an author-based cross-check of all 15 publications authored by Shoichet BK within the final corpus. Fourteen of these publications overlapped with the category-based candidate set, whereas one additional publication was identified. After title and abstract screening, each publication included in the table was assigned to the subaxis that best reflected its primary focus. "Screening source" denotes the route through which the publication was identified. WoS, Web of Science.

Table S3. General characteristics of the bibliometric dataset

Main Information Metrics	Value
Timespan	1980–2026
Sources	328
Documents	1,183
Annual growth rate, 1980-2026*	7.67%
Compound annual growth rate, 1980–2025	9.48%
Compound annual growth rate, 1990–2025	10.15%
Compound annual growth rate, 2000–2025	5.10%
Authors	5,332
Authors of single-authored documents	19
International co-authorship	22.57%
Co-authors per document	6.31
Author keywords (DE)	1,634
References	20,273
Document average age	11.7
Average citations per document	35.21

Categorical data are presented as numbers (n) and percentages (%), and continuous or bibliometric indicators are presented as reported by bibliometrix. *The 2026 data include publications indexed up to June 15, 2026.

Table S4. Missing data analysis of the bibliometric dataset.

Missing Data Metrics	Missing counts	Missing, %
Author	0	0.00
Document type	0	0.00
Journal	0	0.00
Language	0	0.00
Publication year	0	0.00
Science categories	0	0.00
Title	0	0.00
Total citations	0	0.00
Cited references	1	0.08
Affiliation	13	1.10
Abstract	15	1.27
Corresponding author	28	2.37
DOI	72	6.09
Keywords Plus	77	6.51
Author keywords	358	30.26

Missing data are presented as number (n) and percentage (%).

Table S5. Keyword-field coverage according to publication period

Publication period	Documents	Author keyword	Keywords Plus term	At least one of either field	Neither field
1980–1999	75	15 (20.0)	52 (69.3)	53 (70.7)	22 (29.3)
2000–2009	228	110 (48.2)	220 (96.5)	227 (99.6)	1 (0.4)
2010–2017	373	245 (65.7)	354 (94.9)	369 (98.9)	4 (1.1)
2018–2026	507	455 (89.7)	480 (94.7)	500 (98.6)	7 (1.4)
Total	1183	825 (69.7)	1106 (93.5)	1149 (97.1)	34 (2.9)

Values are presented as n (%). "Author keyword" and "Keywords Plus term" indicate documents containing at least one term in the respective field. The 2018–2026 period includes records indexed through June 15, 2026.

Table S6. Web of Science Indexing and 2025 JIF Characteristics of the Ten Most Relevant Sources

Rank	Source title	WoS index	JCR category used	2025 JIF	JIF quartile
1	Antimicrobial Agents and Chemotherapy	SCI-E	Microbiology	4.2	Q2
2	Journal of Antimicrobial Chemotherapy	SCI-E	Infectious Diseases	4.1	Q1
3	Diagnostic Microbiology and Infectious Disease	SCI-E	Infectious Diseases	1.8	Q3
4	International Journal of Antimicrobial Agents	SCI-E	Infectious Diseases	5.0	Q1
5	Antibiotics	SCI-E	Infectious Diseases	5.5	Q1
6	Frontiers in Microbiology	SCI-E	Microbiology	5.8	Q1
7	Journal of Clinical Microbiology	SCI-E	Microbiology	5.9	Q1
8	Microbial Drug Resistance	SCI-E	Infectious Diseases	1.8	Q3
9	PLOS ONE	SCI-E	Multidisciplinary Sciences	2.8	Q2
10	European Journal of Clinical Microbiology & Infectious Diseases	SCI-E	Infectious Diseases	3.2	Q2

Sources were ranked by the number of publications in the study corpus. Journal Impact Factor and quartile data were obtained from the 2025 Journal Citation Reports. For journals assigned to multiple categories, the category used for quartile reporting is shown. SCI-E, Science Citation Index Expanded; JCR, Journal Citation Reports; JIF, Journal Impact Factor.

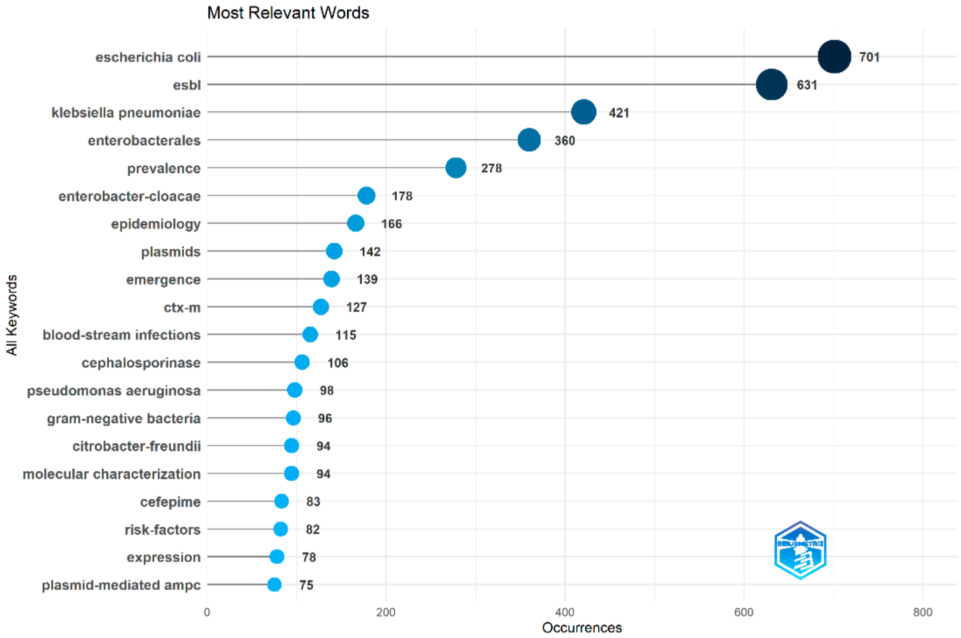

Figure S1. Most relevant keywords in the expanded-search sensitivity analysis. The figure presents the 20 most frequent terms in the "All Keywords" field after synonym standardization and stop-word removal. Circle size and horizontal position indicate the number of occurrences of each term in the expanded-search corpus.

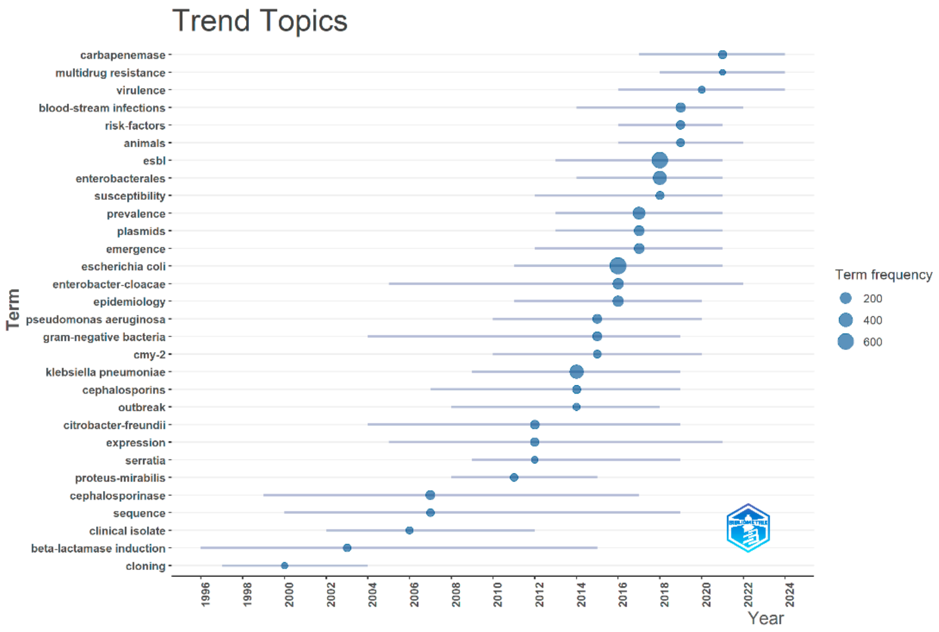

Figure S2. Trend topics in the expanded-search sensitivity analysis. Horizontal lines indicate the temporal span of publications associated with each term, while circles mark the median publication year. Circle size is proportional to term frequency in the expanded-search corpus.

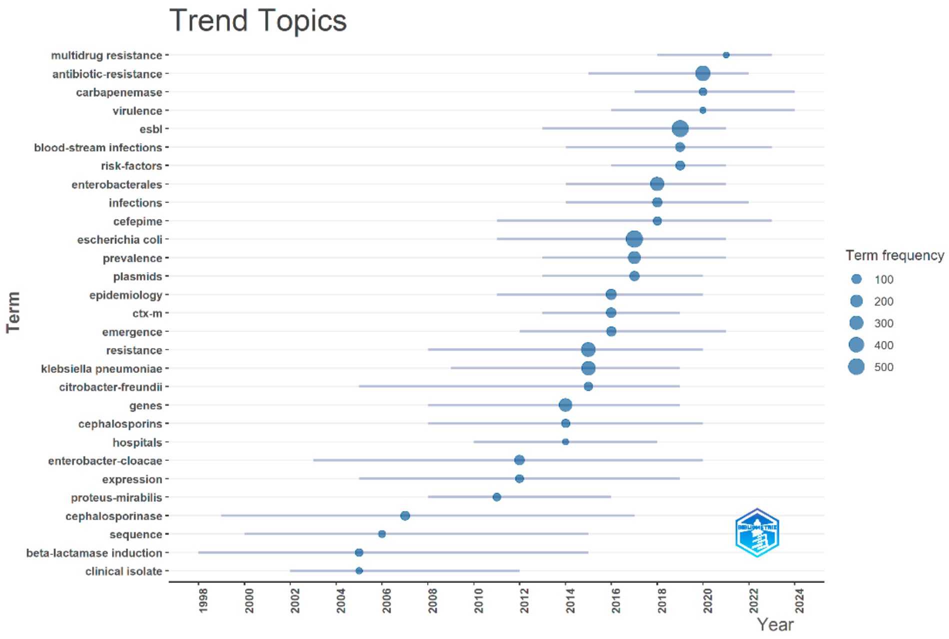

Figure S3. Trend topics in the stop-word sensitivity analysis. Horizontal lines indicate the temporal span of publications associated with each term, while circles mark the median publication year. Circle size is proportional to term frequency in the sensitivity-analysis corpus.

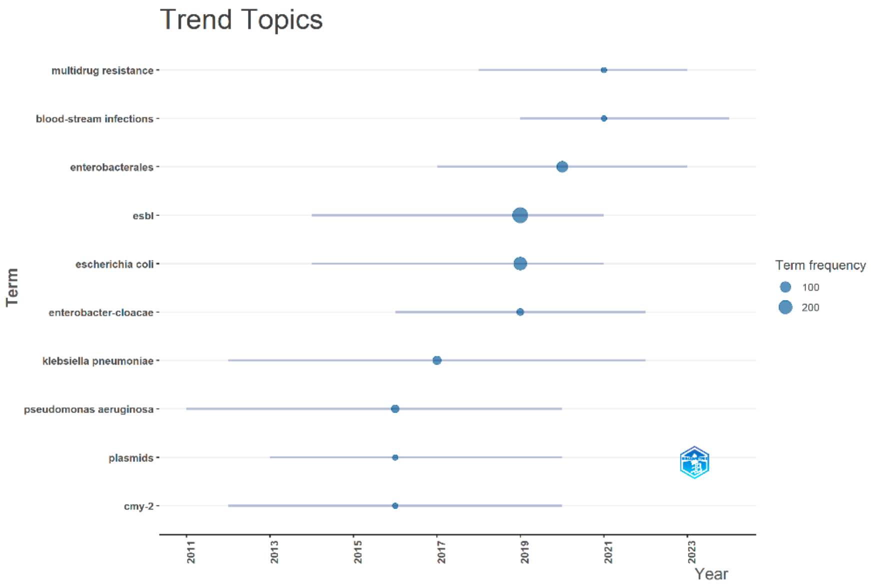

Figure S4. Trend topics in the author-keyword-only sensitivity analysis. Horizontal lines indicate the temporal span of publications associated with each author keyword, while circles mark the median publication year. Circle size is proportional to author-keyword frequency.

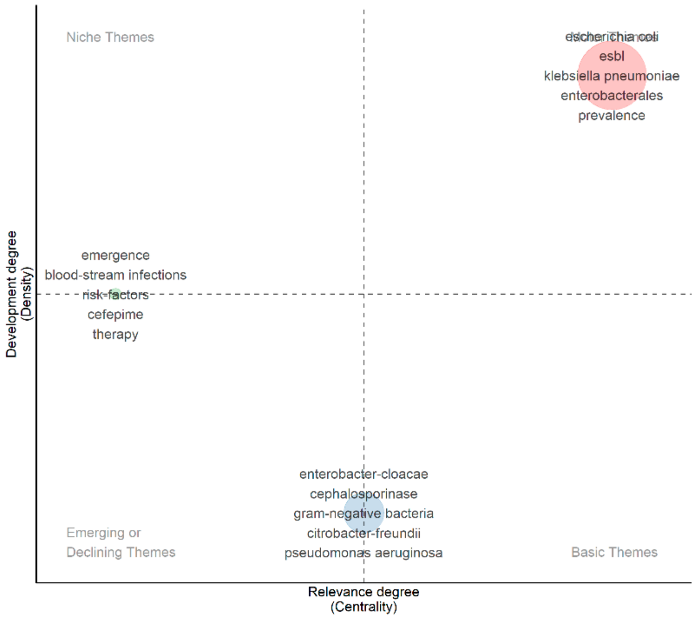

Figure S5. Thematic map in the expanded-search sensitivity analysis. Themes were positioned according to centrality (relevance degree) and density (development degree) and classified as basic, motor, niche, or emerging/declining themes. Bubble size is proportional to cluster frequency, and labels indicate the most representative terms within each thematic cluster.

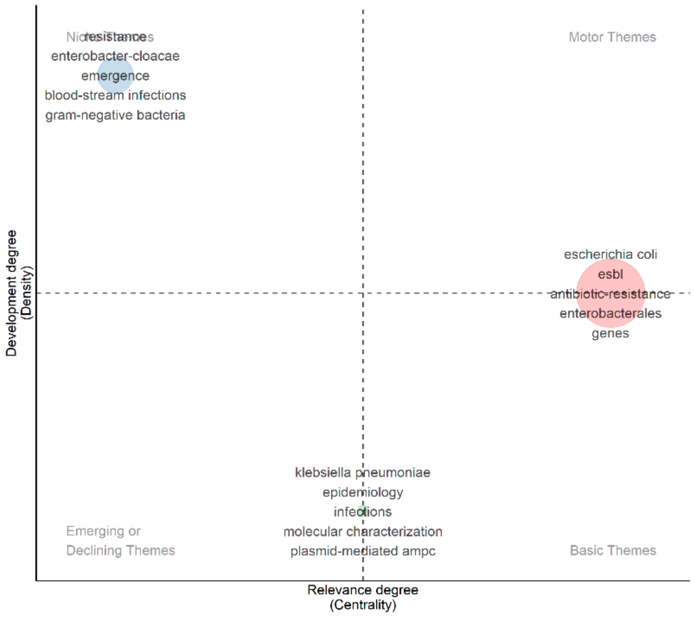

Figure S6. Thematic map in the stop-word sensitivity analysis. Themes were positioned according to centrality (relevance degree) and density (development degree) and classified as basic, motor, niche, or emerging/declining themes. Bubble size is proportional to cluster frequency, and labels indicate the most representative terms within each thematic cluster.

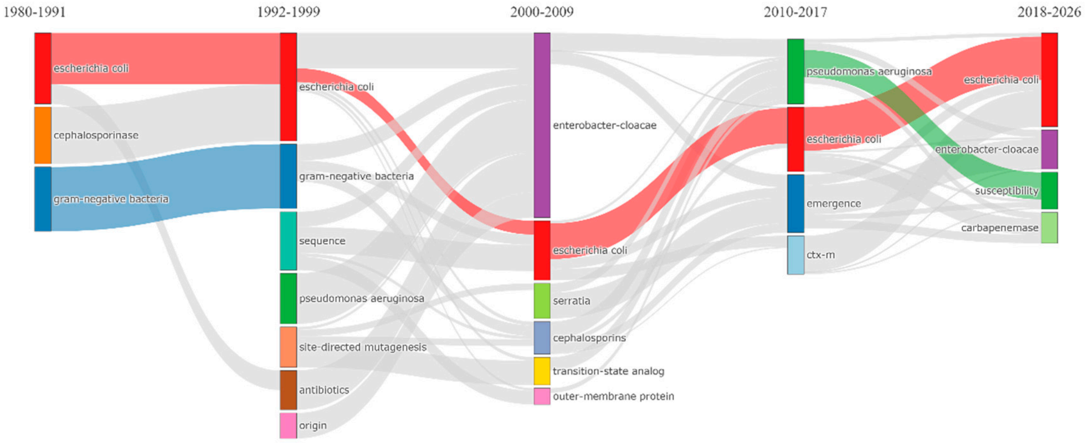

Figure S7. Thematic evolution in the expanded-search sensitivity analysis. The Sankey diagram shows the evolution of thematic clusters across five publication periods: 1980–1991, 1992–1999, 2000–2009, 2010–2017, and 2018–2026. Links indicate thematic continuity or transformation based on shared terms, with wider links representing stronger overlap between consecutive periods.

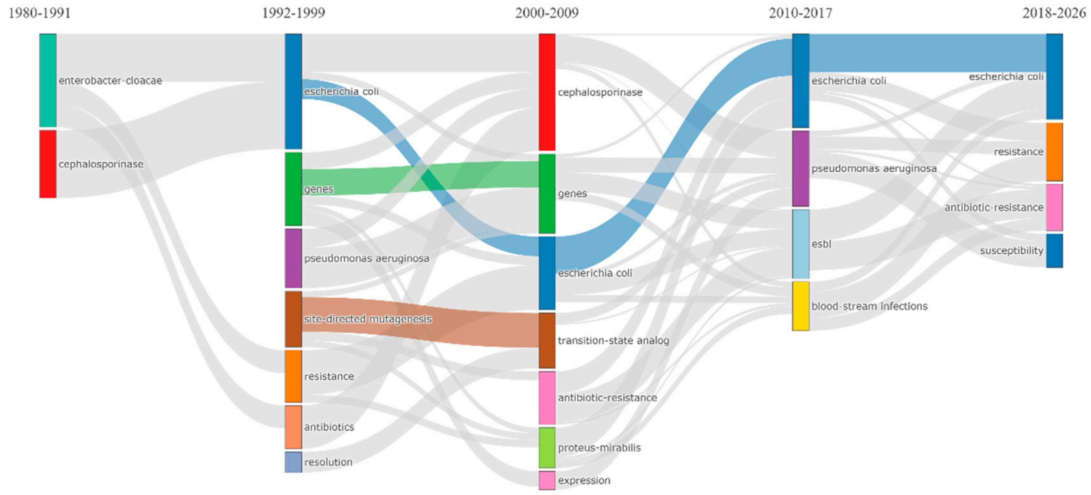

Figure S8. Thematic evolution in the stop-word sensitivity analysis. The Sankey diagram illustrates the evolution of thematic clusters across five publication periods: 1980–1991, 1992–1999, 2000–2009, 2010–2017, and 2018–2026. Links represent thematic continuity or transformation based on shared terms between consecutive periods, with wider links indicating greater thematic overlap.

Supplementary Methods

Trend Topics Analysis Parameters

Trend topics analysis was performed using the "All Keywords" field after synonym standardization and stop-word removal. Owing to the limited keyword coverage in earlier records, the analysis was restricted to publications from 2000 to 2026. Up to 30 terms meeting the predefined minimum frequency threshold were displayed, with a maximum of three terms shown for each year. The analysis was conducted at the unigram level without stemming.

Thematic map and Thematic Evolution Analysis Parameters

The thematic map and thematic evolution analyses were performed after synonym standardization and stop-word removal. A maximum of 100 terms was included in the analysis, and the minimum cluster frequency was set at 8 per 1,000 documents. Analyses were conducted at the unigram level without stemming. Thematic clustering was performed using the Louvain clustering algorithm. The weighting parameters were set to α = 0.5 and a minimum weight index of 0.1. For visualization, the label size was set to 0.3, and a maximum of three labels was displayed

for each cluster. In the thematic map, themes were positioned according to their centrality and density and classified into four quadrants: basic themes, motor themes, niche themes, and emerging or declining themes.

For the thematic evolution analysis, the study period was divided into five time slices: 1980–1991, 1992–1999, 2000–2009, 2010–2017, and 2018–2026. The evolution of themes across these consecutive periods was visualized using a Sankey-type thematic evolution diagram.

Co-occurrence Network Analysis Parameters

Keyword co-occurrence network analysis was performed using the “All Keywords” field after synonym standardization and stop-word removal. The network was normalized using association normalization, and communities were identified using the Louvain clustering algorithm. An automatic network layout was applied, and the network was restricted to 45 nodes. Isolated nodes were removed, and the minimum number of edges required for node retention was set to two. Node coloring by year was not applied, and the repulsion force was set to 0.5. All retained nodes were eligible for labeling, with label scaling enabled and the label size set to 2. For visualization, opacity was set to 0.7 and edge size to 5.

Synonyms List

escherichia coli; escherichia-coli; e coli; escherichia coli; escherichia colt; esherichia coli; e coli; e coil; e -coli; coli; escherichia-coli strains; escherichia coli; clinical escherichia-coli; e. coli

klebsiella pneumoniae; klebsiella-pneumoniae; klebsiella pneumoniae; klebsiella pneumonia; klebsiella pneumoniae; k pneumoniae

klebsiella; klebsiella spp

Enterobacterales; enterobacteriaceae; enterobacteria; family enterobacteriaceae; enterobacteriaceae; Enterobacterales; enterobactenaceae; enterobacteriacea; enterobactriaceae

esbl; esbls; extended-spectrum; spectrum-beta-lactamase; extended-spectrum beta-lactamase; extended-spectrum beta-lactamases; extended spectrum beta-lactamases; extended-spectrum beta-lactamase (esbl); extended spectrum beta-lactamase; extended-spectrum β extended-spectrum-beta-lactamase; extended spectrum beta lactamase; extended-spectrum (3-lactamase, extended-spectrum beta lactamase; extended-spectrum beta-lactamases (esbls); -lactamase (esbls); extended -spectrum; extended spectrum β extended spectrum beta -lactamase; extended spectrum beta lactamase (esbl); extended spectrum beta lactamases; extended spectrum beta lactamases (esbl); extended spectrum beta-lactamase (esbl); extended spectrum beta-lactamases (esbls); extended spectrum p-lactamases (esbls); extended-spectrum -lactamase; extended-spectrum lactamases; extended-spectrumbeta-lactamase (esbl); extended-spectum beta-lactamase; extendedspectrum?-lactamases; spectrum beta-lactamases; spectrum beta-lactamase

beta lactamase; beta-lactamase; beta-lactamases; -lactamase

cefepime; cefepime therapy

gram-negative bacteria; gram-negative bacilli; gram-negative bacterium; gram negative bacteria; gram negative organisms; gram-negative bacterial pathogens; gram-negative organisms; gram-negative rods

molecular characterization; molecular characteristics

plasmids; plasmid

cephalosporinase; cephalosporinase production; cephalosporinases

enterobacter-cloacae; enterobacter cloacae; enterobacter; cloacae; enterobacter cloacae complex

plasmid-mediated ampc; plasmid-mediated ampc beta-lactamase; plasmid-mediated ampc beta-lactamases; ampc beta-lactamase plasmid-encoded; ampc-plasmid mediated

cephalosporins; cephalosporin

pseudomonas aeruginosa; pseudomonas-aeruginosa; pseudomonas aeruginosa

citrobacter-freundii; citrobacter; citrobacter freundii; citrobacter-freundii ampr; citrobacter freundii; citro-bacter freundii; citrobacter freundii complex; citrobacter freundii

cmy; bla(cmy)

cmy-2; bla(cmy-2)

blood-stream infections; bloodstream infection; bloodstream infections; blood-stream infection; bacteremia

proteus-mirabilis; proteus mirabilis; proteus mirabilis

piperacillin-tazobactam; piperacillin/tazobactam

beta-lactamase induction; inducible ampc beta-lactamase; inducible beta-lactamase; inductible beta-lactamases; induction

acinetobacter-baumannii; acinetobacter baumannii

aerogenes; klebsiella aerogenes; klebsiella aerogenes; enterobacter aerogenes; enterobacter-aerogenes

morganella-morganii; morganella morganii; morganella

serratia; serratia marcescens; serratia-marcescens

carbapenemase; carbapenemases

Stop-Words List

antibiotic-resistance

antibiotic resistance

drug resistance

antimicrobial resistance

resistance

ampc

beta lactamase

ampc beta-lactamase

strains

bacteria

spp

ampc beta-lactamases

na

resistant

e

isolate

ampc-beta-lactamase

infections

infection

gene

genes

identification

isolate

isolates

spp.
